# Epiphytes in wooded pastures: Isolation matters for lichen but not for bryophyte species richness

**DOI:** 10.1371/journal.pone.0182065

**Published:** 2017-07-25

**Authors:** Thomas Kiebacher, Christine Keller, Christoph Scheidegger, Ariel Bergamini

**Affiliations:** Department of Biodiversity and Conservation Biology, Swiss Federal Institute for Forest, Snow and Landscape Research WSL, Birmensdorf, Canton of Zürich, Switzerland; University of Guelph, CANADA

## Abstract

Sylvo-pastoral systems are species-rich man-made landscapes that are currently often severely threatened by abandonment or management intensification. At low tree densities, single trees in these systems represent habitat islands for epiphytic cryptogams. Here, we focused on sycamore maple (*Acer pseudoplatanus*) wooded pastures in the northern European Alps. We assessed per tree species richness of bryophytes and lichens on 90 sycamore maple trees distributed across six study sites. We analysed the effects of a range of explanatory variables (tree characteristics, environmental variables and isolation measures) on the richness of epiphytic bryophytes and lichens and various functional subgroups (based on diaspore size, habitat preference and red list status). Furthermore, we estimated the effect of these variables on the occurrence of two specific bryophyte species (*Tayloria rudolphiana*, *Orthotrichum rogeri*) and one lichen species (*Lobaria pulmonaria*) of major conservation concern. Bryophytes and lichens, as well as their subgroups, were differently and sometimes contrastingly affected by the variables considered: tree diameter at breast height had no significant effect on bryophytes but negatively affected many lichen groups; tree phenological age positively affected red-listed lichens but not red-listed bryophytes; increasing isolation from neighbouring trees negatively affected lichens but not bryophytes. However, the high-priority bryophyte species *T*. *rudolphiana* was also negatively affected by increased isolation at small spatial scales. *Orthotrichum rogeri* was more frequent on young trees and *L*. *pulmonaria* was more frequent on trees with thin stems and large crowns. The results indicate that local dispersal is important for lichens, whereas long distance dispersal seems to be more important for colonisation by bryophytes. Furthermore, our study highlights that different conservation measures need to be taken depending on the taxonomic and functional species group or the individual species that is addressed. In practice, for the conservation of a high overall richness in sylvo-pastoral systems, it is crucial to sustain not only old and large trees but rather a wide range of tree sizes and ages.

## Introduction

Sylvo-pastoral systems are old, man-made habitats of great value for nature conservation [1,2], but these systems are presently experiencing serious declines [3,4]. Sylvo-pastoral systems are characterized by high structural heterogeneity and they host a high species richness (richness) [1,3]. In Europe, they are among the most species-rich ecosystems [5,6].

Sylvo-pastoral systems comprise a gradient from forest-like to open landscapes with scattered trees [1,7]. The trees in these systems represent habitat islands for epiphytes, such as bryophytes and lichens, and at low tree densities isolation may lead to poor representation of dispersal-limited species [8]. Compared to trees in closed stands, free-standing trees are subjected to different environmental conditions [4,9]. For example, light levels, temperature and evaporation are much higher at the stem base and in most parts of the outer crown of free-standing trees than of forest trees [9,10].

Bryophytes and lichens are different taxonomic groups. Nonetheless, they are often similar in size and share ecophysiological properties like poikilohydry and possess similar nutrient and water uptake mechanisms, i.e., water and nutrients are absorbed across the entire surface [9]. Consequently they often occur in similar ecological niches [11,12]. Epiphytic bryophyte and lichen richness are thus influenced similarly by a range of variables: the richness of both groups typically increases with increasing tree size, age and stem diameter [13–16], and higher humidity levels usually favour rich bryophyte and lichen communities [17–19]. Increasing solar radiation generally has a positive effect on the richness of epiphytic bryophytes and lichens [20,21] but also a negative effect on humidity [22]. Furthermore, habitat loss and isolation may negatively affect the richness of both species groups and may especially affect species with low dispersal capacities (e.g., species with large diaspores) [23,24]. However, little is known about how all these variables affect richness at the whole-tree scale (per tree richness; i.e., considering the entire bark surface including the tree crown) in sylvo-pastoral systems.

In this study, we focused on sycamore maple (*Acer pseudoplatanus*) wooded pastures because they are rich in bryophyte and lichen species and because they host a number of rare species [25]. Sycamore maple wooded pastures are a traditional land management system in the montane region of the northern Alps. They are dominated by old sycamore maple trees, which are usually found in low spatial densities. The trees in these pastures are the habitat of three species of European conservation concern: the bryophytes *Tayloria rudolphiana* (Garov.) Bruch & Schimp. (Splachnaceae) and *Orthotrichum rogeri* Brid. (Orthotrichaceae) and the lichen *Lobaria pulmonaria* (L.) Hoffm. (Lobariaceae) [2,26]. The globally rare *T*. *rudolphiana* (listed in the IUCN Red List; [27]) is only known to occur in the northern European Alps and in a few sites in China [28]. In Europe, most records are from sycamore maple wooded pastures [29,30]. *Orthotrichum rogeri* is a rare minute moss known to occur in Europe and in a single locality in Asia [30,31]. The lichen *L*. *pulmonaria* has a palearctic distribution and is a flagship species in nature conservation in Europe [32,33]. Like other sylvo-pastoral systems, sycamore maple wooded pastures are severely threatened by ongoing land-use changes such as abandonment as well as management intensification [1,7,34,35]. Both processes are presumed to decrease epiphytic richness: management intensification tends to lead to a decrease in tree densities and thus to larger distances between trees, whereas abandonment may lead to forest ingrowth and to changes in the typical environmental conditions experienced by solitary trees. There is thus an urgent need for deeper knowledge on the drivers of richness in sycamore maple wooded pastures. Furthermore, in contrast to other European sylvo-pastoral systems, like the Fennoscandian wood pastures or the Iberian Dehesas, sycamore maple wooded pastures have so far widely been ignored from biodiversity research and from a conservation perspective [2].

We studied per tree richness of epiphytic bryophytes and lichens on sycamore maple trees, including occurrences within the crown, and we considered richness of several subgroups expected to depend on different drivers. We assessed obligate epiphytes and facultative epiphytes, species with small and large diaspores, red-listed, and non-red-listed species. Furthermore, we included an investigation of the three focal species *T*. *rudolphiana*, *O*. *rogeri* and *L*. *pulmonaria*.

Our objectives are to determine: i) Which variables can explain per tree richness of epiphytic bryophytes and lichens in an open sylvo-pastoral system? ii) Do species that differ in habitat preference (obligate vs. facultative epiphytes), diaspore size (large vs. small) or red-list status (threatened vs. non-threatened) respond differently to these variables? iii) Which variables need to be considered for effective conservation of *T*. *rudolphiana*, *O*. *rogeri* and *L*. *pulmonaria*?

## Methods

### Study sites

**Fig 1:**
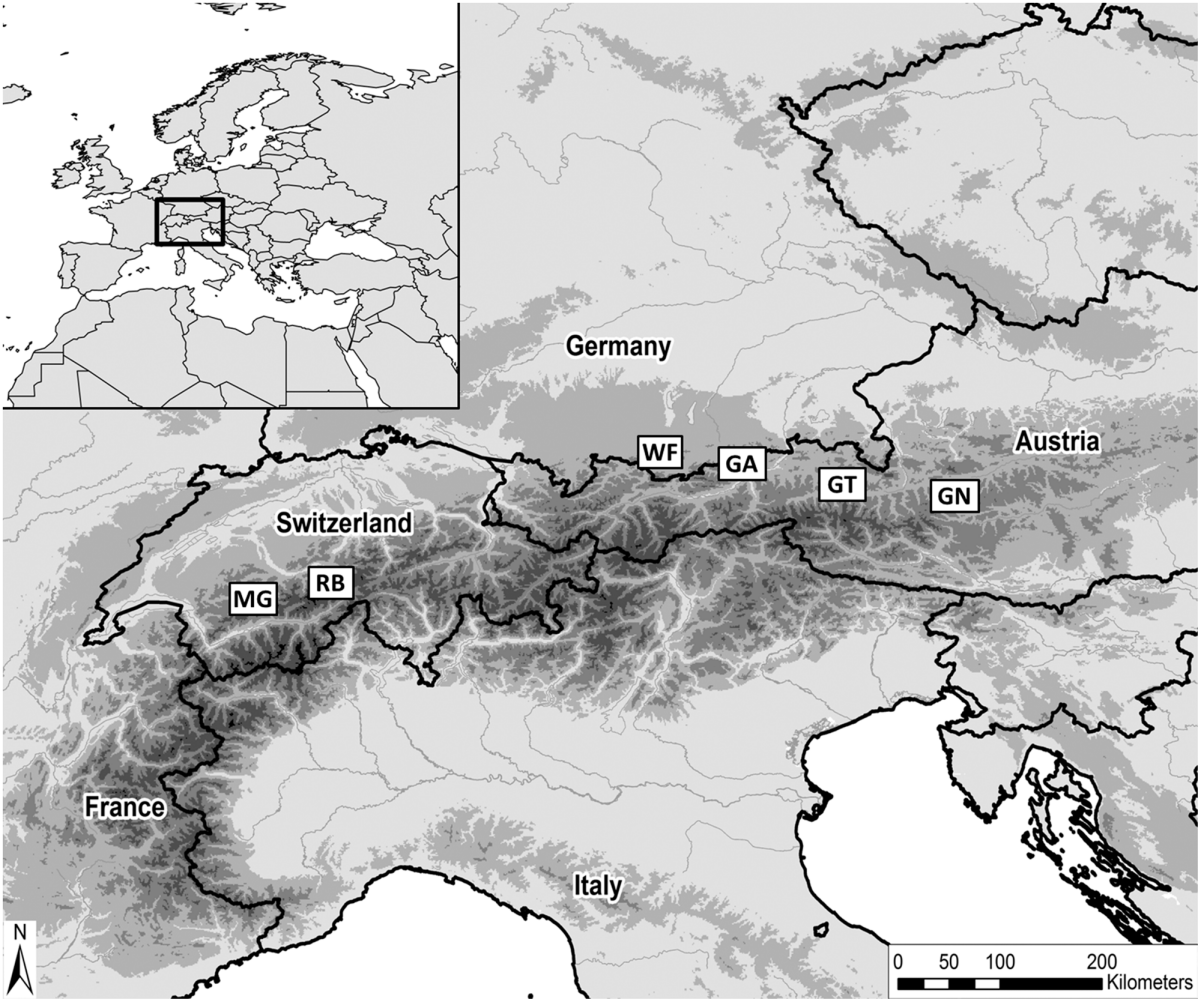
Location of the six study sites in the northern European Alps. RB, Reichenbachtal (Bern, CHE); MG, Meniggrund (Bern, CHE); GA, Grosser Ahornboden (Tyrol, AUT); WF, Wanker Fleck (Bavaria, DEU); GN, Gnadenalm (Salzburg, AUT); GT, Glemmtal (Salzburg, AUT). USGS EROS Data Center; ESRI: ArcWorld Supplement and Data Solutions, B.V.

We selected six study sites, where abundant sycamore maple wooded pastures occur, along the east-west axis of the northern European Alps and we chose three of these sites, Reichenbachtal (RB), Grosser Ahornboden (GA) and Gnadenalm (GN), because of the occurrence of the focal bryophyte species *T*. *rudolphiana* (Fig 1, Fig A in S1 Appendix, Table A in S1 Appendix). Three sites were located in Austria, two in Switzerland, and one in Germany. Within each site, we considered all sycamore maple wooded pastures from ca. 1000 m a.s.l. up to their upper altitudinal boundary at ca. 1700 m a.s.l. The sites are characterized by a temperate mountain climate with high precipitation [36].

### Tree selection

For each site, we digitally marked all sycamore maple trees in pastures on aerial images and we validated tree species identity in the field. Within sites, in order to capture the ecological variability of each site, we applied a stratified random sampling design to select trees for field sampling. For the stratification we used a three-by-two factorial design, in which the three factors were 1) annual global potential shortwave radiation (algorithm following Kumar et al. [37]), to account for different levels of radiation; 2) distance to nearest perennial river with minimum average discharge of ca. 0.25 m^3^/s, to account for different levels of humidity; and 3) stand size (minimum distance between stands = 20 m; for a definition of stands see Kiebacher et al. [25]), to account for different levels of isolation. To avoid spatial aggregation of the sampled trees, only one tree per stand was selected. At each site, interviews with local farmers ("time witnesses", the youngest born in 1933) revealed that the density and distribution of the sycamore maple trees on the pastures had not changed considerably since the 1960’s, thus historic isolation of the individual trees approximates recent isolation. Eighty trees were selected for field sampling by applying the stratified random selection (Table A in S1 Appendix). Further details about the selection procedure are provided in Kiebacher et al. [25]. At GA, GN and RB, we additionally sampled 3–4 trees on which the focal species *T*. *rudolphiana* was found. Within these sites, *T*. *rudolphiana* occurs clustered in spatially segregated subpopulations. We randomly selected one tree per subpopulation (by writing tree numbers on small paper sheets and blindly picking out one sheet). Hence, these additionally selected trees were not spatially aggregated. In total, we examined 90 trees (80 due to the stratification and 10 additional trees with *T*. *rudolphiana*). We only considered trees with a minimum diameter at breast height (DBH; measured at 1.3 m height) of 36 cm, a threshold which is used in in the Swiss National Forest Inventory [38]. For each sampled tree, we measured the DBH, the total height and the height of the lower edge of the crown (defined as the height of the lowest branch). Tree size and DBH are frequently not closely related to tree age [13]. Therefore, we used other proxies based on tree phenology ("phenological age") to classify the age of the trees [25]. We defined trees with smooth stem bark and with a regular branching pattern as young trees, trees with cracked bark and an irregular branching pattern as mature trees and, trees with an especially old appearance (markedly gnarled, large DBH of at least 80 cm, irregular branching pattern and cracked bark far up the tree) as ancient trees. We roughly estimated the volume of the crown as the height of the crown multiplied by the projected crown area. The DBH of the sampled trees ranged from 36 to 168 cm (78.5 ± 29.1, mean ± SD), tree height ranged from 9 to 25 m (16.9 ± 4.0), and crown volume ranged from 122 to 6044 m^3^ (1689 ± 1219). Within a radius of 50 m to each sampled tree, we recorded the DBH of all neighbouring trees with DBH ≥ 36 cm.

### Sampling

Field work was conducted between April and September of 2012 and 2013. We recorded the presence of bryophyte and lichen species in a total of 13 plots per tree. We collected species that could not be identified in the field and examined them in the lab. Protected species were identifiable in the field, not collected and hence, not affected. We selectively placed the plots on different microhabitats of the tree (Table 1, Fig B in S1 Appendix). The sampled area per tree ranged from 3.5 to 13.3 m^2^ (6.5 ± 2.2, mean ± SD).

**Table 1:** Sampling design applied to record per tree richness of bryophytes and lichens on sycamore maple trees.

Microhabitat	Plot placement	Plot size, shape	No. plots
**Tree base**	On the stem, from ground level up to a height of 0.5 m (including major roots above the soil surface)	truncated cone-shaped, height 0.5 m	1
**Stem below the crown**	On the stem, with the top of the plot at the lowest branch and extending 0.75 m downwards	Cylindrical, height 0.75 m	1
**Crutches**	In the major crutches	Rectangular, size varying according to anatomy of the crutch	2
**Largest branches**	On the largest branches or on the stem within the crown	Cylindrical, length 0.6 m	3
**Medium-sized branches**	On branches of intermediate thickness	Cylindrical, length 0.6 m	3
**Thin branches**	On thin branches (up to 5 cm in diameter) in the outer crown	Cylindrical, length 0.6 m	3

All bryophyte and lichen species present within each plot were recorded. For an example of the placement of the plots, see Fig B in S1 Appendix.

We climbed trees to assess plots > 2 m above ground. To sample the outer crown, we cut thin branches by using a 6 m long telescopic tree pruner. The sampling procedure aimed at maximizing the number of bryophyte species recorded for each tree. First, we surveyed the tree base and the stem below the crown. We then placed the plots in the microhabitats within the crown (crutches, largest branches, medium sized branches) so that additional bryophyte species were represented in these plots. We did not look for additional lichen species to place the plots. Hence, for lichens the sampling was non-selective. If no additional bryophyte species was found, we tried to cover the structural and ecological variability of the corresponding microhabitat with the remaining plots. We also used this latter criterion to select the thin branches that were cut to get a complete as possible list of species per tree. By applying this sampling procedure, we could record almost all bryophyte species and a representative number of lichen species per tree, which was ascertained by species accumulation curves [25]. Further details about the study sites and the sampling procedure are provided in Kiebacher et al. [25].

### Nomenclature

The nomenclature for bryophytes follows Hill et al. [39] and Söderström et al. [40,41]. The nomenclature for lichens follows Clerc and Truong [42] and, for species not included in that publication, Wirth et al. [43] and Saag et al. [44]. Some closely related species that are difficult to identify were treated as aggregates, as defined by Kiebacher et al. [25].

### Definition of species groups

We classified species into obligate epiphytes ('epiphytes'), i.e., species that usually grow on the bark of living trees and shrubs in the study area, and facultative epiphytes ('non-epiphytes'), i.e., species that usually grow on other substrates. These classifications were based on Clauzade et al. [45], Frahm and Frey [46], Nebel and Philippi [47–49], Ignatova and Ignatov [50], Wirth et al. [43] as well as on our field experience in the study region. Two taxa remained unclassified: *Bacidia arnoldiana* aggr. comprised epiphytes and non-epiphytes and *Bacidia viridifarinosa* was not classified because of uncertain identification and possible confusion with species with different habitat preferences (Table B in S1 Appendix). We defined red-listed species as species with red-list status critically endangered (CR), endangered (EN) or vulnerable (VU) according to Schnyder et al. [51] for bryophytes and Scheidegger et al. [52] for lichens. To classify bryophytes and lichens into species with small and large diaspores, we considered the size of the prevalent diaspore type: for species which regularly reproduce sexually we considered the spores and for species which do not or only occasionally reproduce sexually, we considered the asexual propagules (deciduous branchlets and leaves, gemmae, bulbils, soredia, isidia). We used a threshold of 50 μm to distinguish between small and large diaspores. Diaspore sizes were derived from literature [43,53–55]. We chose this threshold because most of the surveyed bryophyte and lichen species had spores smaller than 50 μm, whereas their asexual propagules (e.g., deciduous branches or isidia) are usually larger (see [54,56]). Thus, species with small diaspores mainly comprise species that regularly reproduce sexually, and species with large diaspores mainly comprise species which are dispersed by asexual propagules. Four taxa remained unclassified because of uncertain identification (Table B in S1 Appendix).

### Analyses

We analysed the data using R, version 3.2.1 [57]. To relate the richness of bryophytes and lichens to a range of explanatory variables, we constructed generalized linear mixed effects models (GLMM; [58]) using the "lme4" package in R [59]. We specified a Poisson error structure and a log-link function. To analyse the occurrence of the three focal species, we used GLMM's with a binomial error structure and a logit-link function.

As random effects, we considered study site and a two-level factor that specified whether a tree was randomly selected or one of the additionally selected trees (with occurrences of *T*. *rudolphiana*). The latter factor was nested within site. For the *T*. *rudolphiana* model, we did not consider this factor and used 'site' as the only random intercept effect. As fixed effects, we considered a set of tree characteristics, environmental variables and isolation measures which were estimated for each sampled tree. In total, the initial predictor set consisted of 15 variables (Table C in S1 Appendix). We tested the initial predictor set for collinearities among predictors and we chose a threshold value of |Spearman's rho| > 0.7 to exclude highly correlated predictors (see correlation table in Table D in S1 Appendix). The final predictor set is given in Table 2. To increase the comparability of the parameter estimates, we standardized all numeric predictors to a mean of 0 and a standard deviation of 0.5 [60,61].

**Table 2:** Final predictor set considered as fixed effects for the GLMM analyses.

Predictor	Description	Range	Mean ± SD	Unit
Tree characteristics				
**DBH**	Tree diameter at breast height (1.3 m)	36–168	78.5 ± 29.1	cm
**Crown volume**	Volume of the crown	122–6044	1689 ± 1219	m^3^
**Phenological age**	Three-level factor: 1 young (16.7%), 2 mature (71.1%), 3 ancient (12.2%)			
Environmental variables				
**Altitude**	Altitude above sea level	1048–1529	1276 ± 115	m
**Distance to river**	Minimum Euclidian distance to nearest river	2–3330	424 ± 695	m
**Radiation**	Annual global potential shortwave radiation (algorithm following Kumar et al. [37])	9964–22358	15736 ± 2256	kJ m^-2^ day^-1^
Isolation measures				
**Pot.habitat50m**	Potential habitat amount within a radius of 50 m: Σ DBH_i_^0.6^ of trees within a radius of 50 m (sensu Moilanen and Nieminen [62]; they used Σ Area_i_^0.3^; because DBH is linear we used 0.6 instead of 0.3 as the exponent)	0–540	67.1 ± 93.2	cm^0.6^
**No.trees200m**	Number of neighbouring trees within a radius of 200 m	0–281	44.0 ± 56.3	count

A log transformation was applied to "distance to river" and a square root transformation was applied to "Pot.habitat50m" and "No.trees200m" (detailed descriptions of all predictor variables are provided in the table).

Initially we also included all possible two-way interactions between the variables within the three predictor sets. We ultimately excluded them, however, because they either did not or only marginally improved model fits (in terms of AICc and R^2^ for GLMMs [63]). We checked the full final models, containing all the selected predictors (Table 2), for overdispersion by using the function "dispersion_glmer" implemented in the R-package ''blmeco'' [64]. If necessary (dispersion > 1.4), we accounted for overdispersion by adding the observational level (a factor with an individual level for each tree) as a random intercept effect to the model [64]. However, overdispersion was rare and adding the specified random term successfully reduced dispersion (see Table E in S1 Appendix).

Model simplification and model selection were performed using a multi-model inference framework [65]. Using the "MuMIn" package [66], we generated all sub models including all possible combinations of the predictors and ranked them by AICc. Following Grueber et al. [67], we retained all models which had a Δ AICc < 2 relative to the best model. We applied model averaging to this candidate model set to produce conditional averaged parameter estimates and relative variable importance (RVI) estimates for each predictor [65]. To visualize the effect size of selected predictors, we drew effect plots by holding other predictors constant at their mean, setting phenological age at the level of mature trees, and adding the intercept estimate for the population mean (the average intercept across all sites as returned by the model output) to the fitted values.

## Results

### Richness

We recorded 176 bryophyte and 232 lichen species on the 90 trees (Table 3, see complete species list in Table B in S1 Appendix). Most of the lichen species (202) were epiphytes, whereas most of the bryophyte species (135) were non-epiphytes. Ten bryophyte species (7 of which were epiphytes) and 41 lichen species (all of which were epiphytes) are red-listed species. For bryophytes, the number of species with large diaspores and the number of species with small diaspores were nearly identical, whereas 84% (194 species) of the lichen species had small diaspores (Table 3).

**Table 3:** Total number of species, as well as the minimum, maximum, mean and standard deviation of per tree richness of bryophytes and lichens on 90 sycamore maple trees.

Species group	Bryophytes + Lichens					Bryophytes					Lichens				
	Total	Min	Max	Mean	SD	Total	Min	Max	Mean	SD	Total	Min	Max	Mean	SD
All species	408	42	108	73.02	11.45	176	13	60	28.84	9.13	232	25	67	44.18	7.10
Red-listed	51	1	9	4.18	1.88	10	0	4	1.60	0.95	41	0	7	2.58	1.75
Not red-listed	357	38	102	68.84	10.77	166	13	57	27.24	8.78	191	25	61	41.60	6.44
Small diaspores	279	34	74	55.40	7.48	85	8	32	18.07	5.12	194	21	57	37.33	6.18
Large diaspores	125	8	34	17.11	5.96	87	4	26	10.71	4.72	38	2	12	6.40	2.63
Non-epiphytes	163	2	44	15.18	8.48	135	1	40	12.00	7.56	28	0	9	3.18	1.95
Epiphytes	243	40	78	56.61	6.91	41	9	26	16.49	3.08	202	24	63	40.12	6.69
- Red-listed	48	1	9	4.12	1.87	7	0	4	1.54	0.95	41	0	7	2.58	1.75
- Not red-listed	195	36	71	52.49	6.31	34	9	24	14.94	2.71	161	24	57	37.54	6.04
- Small diaspores	201	31	66	45.83	6.31	27	4	16	10.53	2.24	174	20	55	35.30	6.08
- Large diaspores	42	4	16	10.76	2.42	14	3	10	5.96	1.53	28	1	9	4.80	1.91

The per tree richness ranged from 42 to 108 with a mean of 73.0 ± 11.4 (SD). The number of bryophyte species per tree ranged from 13 to 60 (28.8 ± 9.1) and that of lichen species ranged from 25 to 67 (44.2 ± 7.1). The values for the subgroups are given in Table 3.

We recorded *T*. *rudolphiana* on 16 of the 90 sampled trees (on six randomly selected trees) and we did not find the species at MG, WF and GT. *Lobaria pulmonaria* was present on 39 trees, and the site MG was the only site where we did not record *L*. *pulmonaria* on the sampled trees (but we observed the species on neighbouring trees). We recorded *O*. *rogeri* on 29 trees distributed across all six sites.

### Drivers of epiphyte richness

Variables from all three predictor groups (tree characteristics, environmental variables and isolation measures) affected epiphyte richness, and bryophytes and lichens were differently affected by the variables (Table 4, Table E in S1 Appendix).

**Table 4:** Results of the GLMM analyses determining effects on per tree richness of epiphytic bryophytes and lichens.

	Tree characteristics							Environment						Isolation			
	DBH		Crown volume		Phen.age2	Phen.age3		Altitude		Distance to river		Radiation		Pot.habitat50m		No.trees200m	
	Estimate	RVI	Estimate	RVI	Estimate	Estimate	RVI	Estimate	RVI	Estimate	RVI	Estimate	RVI	Estimate	RVI	Estimate	RVI
**Bryophytes**																	
All species			0.10*	**0.81**	0.20**	0.28**	**1.00**	**0.12**#	**0.72**	-0.05ns	0.17	-0.10*	**1.00**				
Red-listed	0.24ns	0.37	0.13ns	0.15						0.43*	**0.82**			-0.23ns	0.28	-0.24ns	0.29
Not red-listed			**0.09**#	**0.74**	0.22***	0.31***	**1.00**	0.09ns	0.41	-0.06ns	0.23	-0.11*	**1.00**			-0.04ns	0.09
Small diaspores	0.06ns	0.30	0.14*	**1.00**								-0.03ns	0.12			-0.12ns	0.60
Large diaspores			0.06ns	0.16	0.45***	0.62***	**1.00**	0.12ns	0.24	-0.09ns	0.21	-0.20**	**1.00**				
Non-epiphytes					0.52**	0.81***	**1.00**	0.28ns	0.46	-0.12ns	0.20	**-0.23**#	**0.77**	-0.14ns	0.10		
Epiphytes	0.07ns	0.20	0.07ns	0.19				-0.15*	**0.86**	0.04ns	0.10					-0.05ns	0.11
- Red-listed	0.22ns	0.22	0.14ns	0.09				0.13ns	0.08	0.45*	**0.83**			-0.21ns	0.13	-0.25ns	0.18
- Not red-listed	0.06ns	0.21	0.07ns	0.24				-0.18**	**1.00**							-0.04ns	0.16
- Small diaspores			0.04ns	0.15				-0.12ns	0.27	0.08ns	0.20						
- Large diaspores	**0.15**#	**0.37**	0.12ns	0.09	0.27*	**0.32**#	**0.35**	-0.23*	**1.00**	-0.08ns	0.14	0.09ns	0.14			-0.09ns	0.21
**Lichens**																	
All species	-0.05ns	0.41	-0.04ns	0.31				0.10*	**0.52**	-0.04ns	0.20	-0.03ns	0.06			0.14***	**1.00**
Red-listed	-0.76***	**1.00**			**0.38**#	1.05**	**1.00**	-0.12ns	0.26			-0.10ns	0.13	-0.28ns	0.40	0.44*	**1.00**
Not red-listed	-0.05ns	0.17	-0.05ns	0.15				0.10**	**0.78**	-0.04ns	0.17	-0.02ns	0.08	0.04ns	0.09	0.11**	**1.00**
Small diaspores	**-0.07**#	**0.65**	-0.06ns	0.36						**-0.08**#	**0.79**					0.11*	**1.00**
Large diaspores	-0.13ns	0.35			0.37*	0.54**	**1.00**	0.39***	**1.00**	0.23*	**1.00**					0.27**	**1.00**
Non-epiphytes	0.33*	**0.74**	-0.17ns	0.16	0.46*	0.60*	**0.33**	0.38*	**0.95**	0.29*	**0.71**	-0.15ns	0.23			0.17ns	0.13
Epiphytes	-0.09*	**1.00**						0.08*	**0.49**	-0.05ns	0.39					0.14***	**1.00**
- Red-listed	-0.76***	**1.00**			**0.38**#	1.05**	**1.00**	-0.12ns	0.26			-0.10ns	0.13	-0.28ns	0.40	0.44*	**1.00**
- Not red-listed	**-0.06**#	**0.81**						0.10**	**0.55**	-0.05ns	0.34			0.06ns	0.18	0.11*	**0.91**
- Small diaspores	-0.09*	**1.00**								**-0.08**#	**0.65**					0.11*	**1.00**
- Large diaspores	**-0.24**#	**0.31**			**0.30**#	**0.48**#	**0.52**	0.31**	**1.00**	0.17ns	0.51					0.35***	**1.00**

Standardized coefficient estimates, level of significance (in bold: *p* < 0.1) and relative variable importance (RVI) after conditional model averaging are shown for the effects of tree characteristics, environmental variables and isolation measures. All red-listed lichens were epiphytes. DBH, diameter at breast height; Phen.age, phenological age (1 = young trees, 2 = mature trees, 3 = ancient trees; we used the first level of this factor as the baseline for effects of phenological age); Pot.habitat50m, potential habitat amount within a radius of 50 m, No.trees200m, number of trees within a radius of 200 m. Complete results including unconditional standard errors, 95% confidence intervals and *p*-values for each coefficient estimate are provided in Table E in S1 Appendix. ns, not significant (*p* > 0.1),

^#^ *p* < 0.1,

* *p* < 0.05,

** *p* < 0.01,

*** *p* < 0.001.

### Tree characteristics

**Fig 2:**
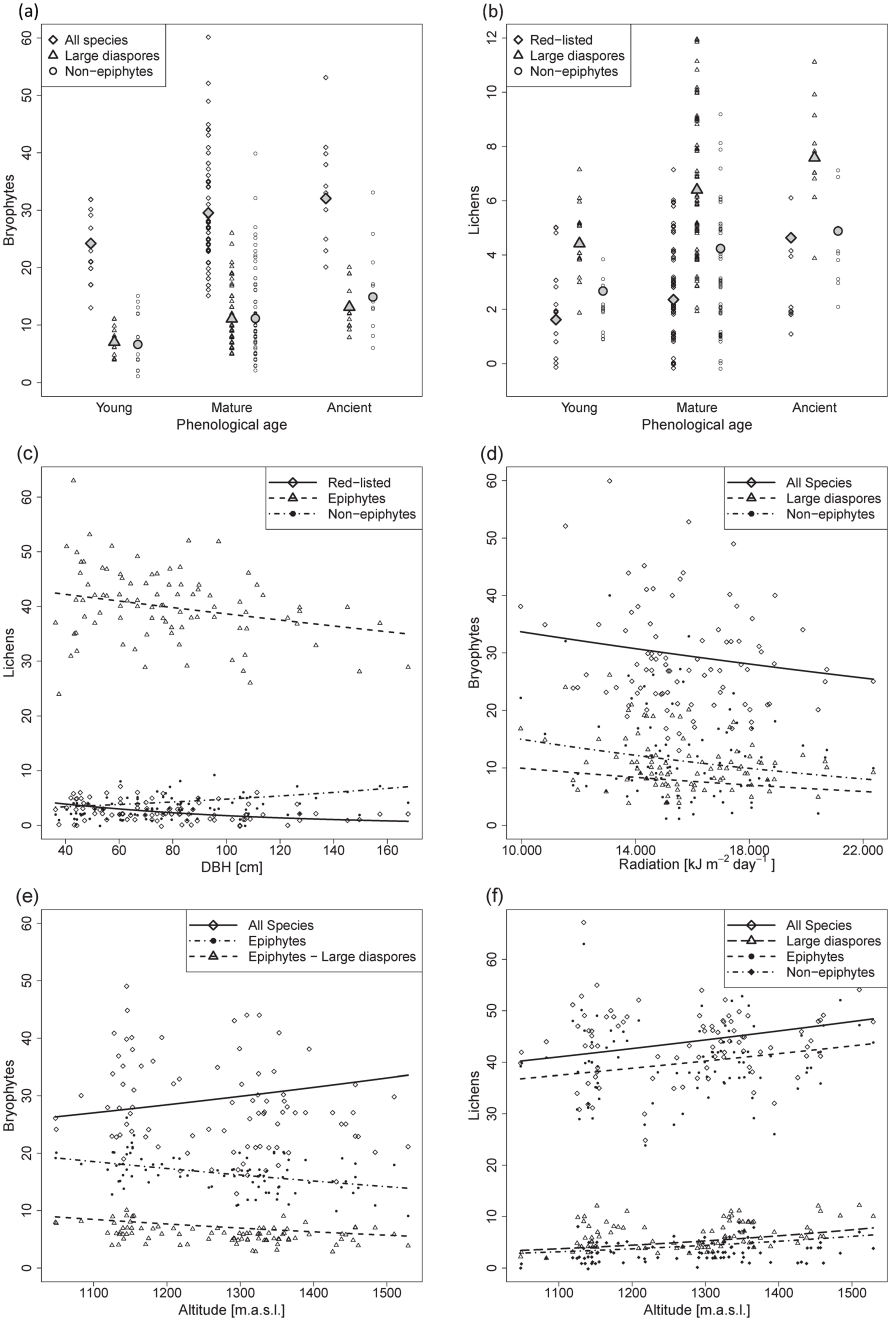
Effects of tree characteristics and environmental variables on epiphyte richness. Effect plots for significant (*p* < 0.05, significance levels according to GLMM analyses; see Table 4) and marginally significant (*p* < 0.1) relationships between (a-b) phenological age, (c) diameter at breast height (DBH), (d) radiation and (e-f) altitude and per tree richness of bryophytes and lichens. Large symbols and regression lines represent the estimated values for the population mean, small symbols represent the observations, i.e., the richness on each sampled tree.

Diameter at breast height did not significantly (*p* > 0.1) affect the richness of bryophytes but affected the richness of many of the lichen groups (Table 4, Fig 2). We found opposing trends for the effect of DBH on lichen epiphytes and non-epiphytes. Epiphytes (*p* = 0.024) and especially red-list species (*p* < 0.001) were significantly more diverse on trees with a small DBH and non-epiphytes (*p* = 0.017) were significantly more diverse on trees with a large DBH. The crown volume was not significantly related to lichen richness, but it had a positive effect on total bryophyte richness (*p* = 0.047) and on bryophytes with small diaspores (*p* = 0.019). For bryophytes as well as for lichens, older phenological age of the trees had a significant positive effect on the richness of non-epiphytes (mature and ancient compared to young: *p* = 0.002 and < 0.001 for bryophytes; *p* = 0.025 and 0.026 for lichens) and of species with large diaspores (*p* = 0.044 and 0.069 for bryophytes; *p* = 0.011 and 0.007 for lichens) but had no significant effect on the richness of epiphytes and of species with small diaspores. Furthermore, the richness of red-listed lichen species (all of which were epiphytes) was significantly higher (*p* = 0.003) on ancient than on young trees and tended to be higher (*p* = 0.094) on mature than on young trees.

### Environment

Richness of all bryophytes and lichens was positively correlated with higher altitudes (Table 4, Fig 2). However, for bryophytes this effect was only marginally significant (*p* = 0.087). For lichens, the effect was only significant (*p* < 0.001) to the small subgroup of species with large diaspores. We did not detect a significant altitudinal pattern for lichen species with small diaspores, which comprised a major part of the species set (Table 3). Considering only the epiphytes, we detected opposing altitudinal trends for bryophytes and lichens. Lichen-epiphytes were more diverse at high altitudes, whereas bryophyte-epiphytes were more diverse at low altitudes. Larger distances to the nearest river positively influenced the richness of red-listed bryophyte species and non-epiphyte-lichens. The distance to the nearest river also had opposing effects on the richness of lichens with contrasting dispersal strategies. Lichen species with large diaspores were more diverse at larger distances, whereas species with small diaspores tended to be more diverse near rivers. Radiation only affected the richness of bryophytes. The richness of all bryophyte species (*p* = 0.024) and of bryophytes with large diaspores (*p* = 0.007) was significantly reduced at higher levels of radiation (Table 4, Fig 2).

### Isolation

**Fig 3:**
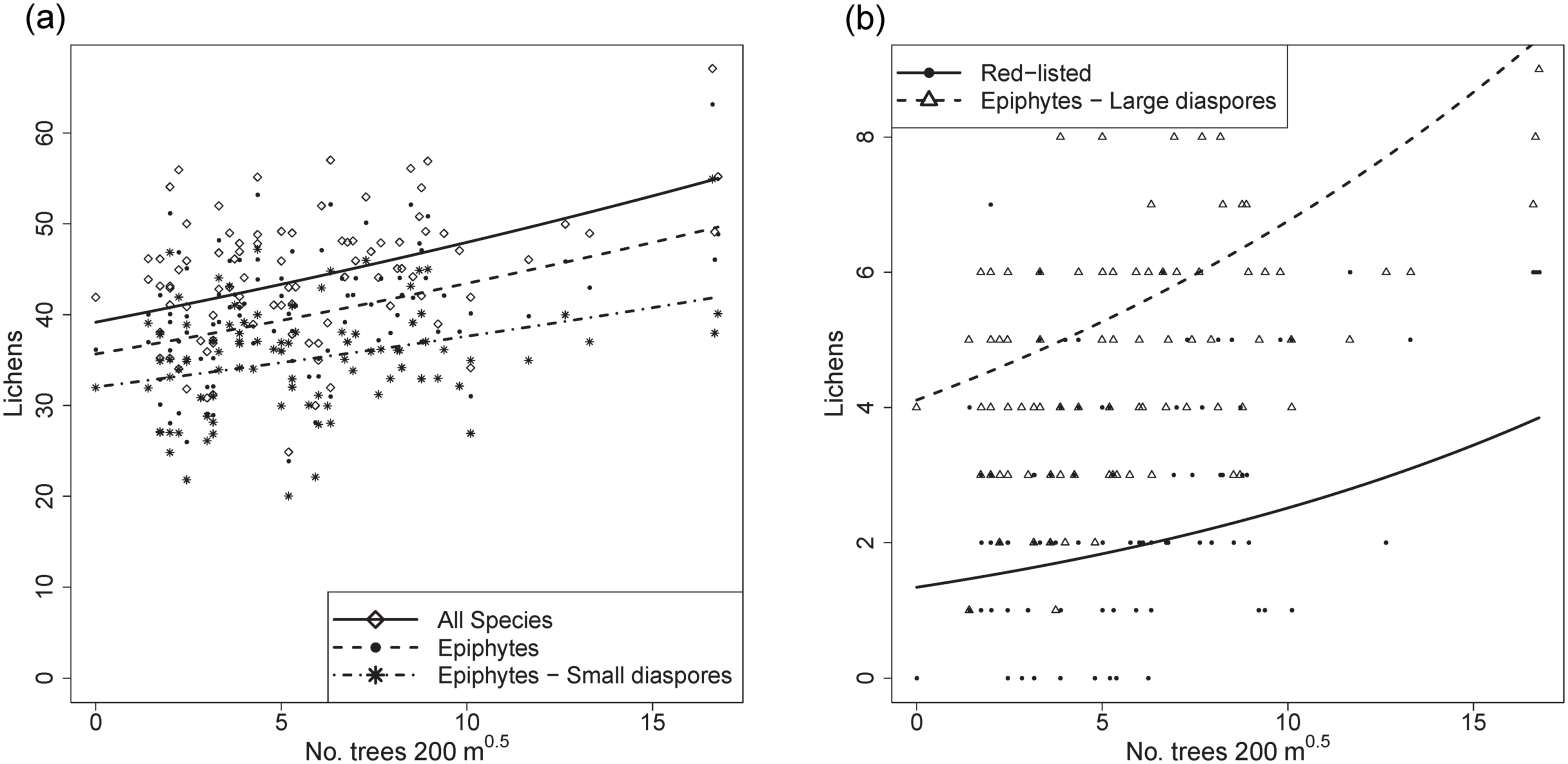
Effects of isolation on epiphyte richness. Effect plots for significant (*p* < 0.05, significance levels according to GLMM analyses; see Table 4) relationships between (a-b) the number of trees within a radius of 200 m (No. Trees 200 m) and the per tree richness of lichens. Regression lines represent the estimated values for the population mean, symbols represent the observations, i.e., the richness on each sampled tree.

We did not found a significant effect of the isolation measures for the richness of bryophytes or any bryophyte subgroup. In contrast, for lichens, the number of trees within a radius of 200 m was significantly positively related to the richness of all species (*p* < 0.001) and to that of all subgroups, with non-epiphytes as the only exception (Table 4, Fig 3). For total lichen richness, as well as for most of the lichen subgroups, isolation was the most important predictor.

### Drivers of the occurrence of the focal species

*Tayloria rudolphiana* was more frequent at high altitudes (*p* = 0.035), at low radiations (*p* = 0.081) and at high amounts of potential habitat within a radius of 50 m (*p* = 0.037, Table 5). *Orthotrichum rogeri* was significantly (*p* = 0.008) more frequent on young than on mature trees and *L*. *pulmonaria* was more frequent on trees with small DBH's (*p* = 0.006) and on trees with large crowns (*p* = 0.023).

**Table 5:** Results of the GLMM analyses determining effects on the occurrence of the three focal species.

Species	Tree characteristics							Environment						Isolation			
	DBH		Crown volume		Phen.age2	Phen.age3		Altitude		Distance to river		Radiation		Pot.habitat50m		No.trees200m	
	Estimate	RVI	Estimate	RVI	Estimate	Estimate	RVI	Estimate	RVI	Estimate	RVI	Estimate	RVI	Estimate	RVI	Estimate	RVI
***T*. *rudolphiana***	**2.18**#	**0.55**	0.99ns	0.15	19.2ns	20.8Ns	0.64	2.90*	**1.00**	-1.21ns	0.12	**-2.13**#	**1.00**	2.41*	**1.00**		
***O*. *rogeri***	1.23ns	0.21	1.16ns	0.33	-2.72**	**-2.79**#	**1.00**			**1.51**#	**0.69**	0.92ns	0.37				
***L*. *pulmonaria***	-3.54**	**1.00**	2.47*	**1.00**	21.4ns	21.7ns	1.00			0.85ns	0.21	-0.61ns	0.17			0.92ns	0.20

Standardized coefficient estimates, level of significance (in bold: *p* < 0.1) and relative variable importance (RVI) after conditional model averaging are shown for the effects of tree characteristics, environmental variables and isolation measures on the occurrence of *Tayloria rudolphiana*, *Orthotrichum rogeri* and *Lobaria pulmonaria*. DBH, diameter at breast height; Phen.age, phenological age (1 = young trees, 2 = mature trees, 3 = ancient trees; we used the first level of this factor as the baseline for effects of phenological age); Pot.habitat50m, potential habitat amount within a radius of 50 m, No.trees200m, number of trees within a radius of 200 m. Complete results including unconditional standard errors, 95% confidence intervals and *p*-values for each coefficient estimate are provided in Table F in S1 Appendix. ns, not significant (*p* > 0.1),

^#^ *p* < 0.1,

* *p* < 0.05,

** *p* < 0.01,

*** *p* < 0.001.

## Discussion

The high richness observed emphasize the particular importance of sycamore maple wooded pastures for bryophyte and lichen conservation. One hundred and seventy bryophyte species (98 genera) and 231 lichen species (79 genera) were present on a total of 90 sycamore maple trees. In a study in Gotland (Sweden), where 1148 trees belonging to 13 deciduous and two coniferous species were examined, a similar number of lichen species was found [68], highlighting the extraordinary richness of sycamore maple wooded pastures. The high richness in sycamore maple wooded pastures may be explained by a humid environment [17–19], sampled trees were old or ancient [14,15], and, compared to large parts of Europe, a low impact from atmospheric pollutants at the end of the 20th century in the study region [25]. Additionally, the extensive sampling design, which included tree crowns, contributed to the high richness reported here. Tree climbing techniques are rarely applied in Europe [69], and there is considerable bias with respect to the intensity with which the different vertical zones of trees have been studied: typically, only the trunks of trees, up to a height of two meters or even less, are sampled (e.g., [70,71]). However, Kiebacher et al. [25] showed that, on average, 60% of lichen species and 30% of bryophyte species are overlooked per tree in the study system if only stems are sampled and very similar numbers were reported from beech- and coniferous forests in Germany [69]. Furthermore, sampling several stems at a site fails to effectively reduce the number of overlooked species [25,69].

Despite similarities in a number of important traits (e.g., size, poikilohydry, small diaspores), the richness of bryophytes and lichens as well as that of the subgroups (epiphytes / non-epiphytes, red listed / not red-listed species, species with small / large diaspores) was affected differently by the predictors. This could be explained by different life strategies and different establishment constraints related to the symbiotic nature of lichens. Different richness patterns of bryophytes and lichens have also been observed in other habitats, e.g., on forest soils [72].

### Tree characteristics

Measures of tree size such as DBH and tree height are known to be important variables for the richness of epiphytic bryophytes and lichens [13,21,32,68]. DBH is frequently used as a proxy for tree age (e.g., [32]), but, this can be misleading [13]. Hence, we estimated tree age using a combination of phenological characteristics that we expected to be more strongly related to tree age than DBH. Our results indicate that it is crucial to distinguish between (phenological) tree age and size-related variables, such as DBH or crown volume, because they can show opposing effects on richness. The generally weak effect of crown volume, and for bryophytes also of DBH, compared to the strong effect of phenological age for both groups indicates that tree age is more important than tree size in supporting high richness. This might be related to higher microhabitat diversity (e.g. crutches with mold accumulation, stem cavities and damages) on older trees [73]. Surprisingly, DBH was negatively related to the richness of red-listed lichens and epiphyte-lichens. It seems likely that bark characteristics that depend on DBH are responsible for this relationship. Independent of age, trees with smaller diameters usually have fewer bark fissures and the rate of scaling is lower in slowly growing (sycamore maple) trees [9]. Bark fissures and loose bark have previously been found to negatively affect lichen richness [9,20]. Additionally, bark fissures favour the establishment of a moss cover [9,74,75]. Thus, on thick-stemmed trees with cracked bark, competing bryophytes might contribute to lower lichen richness. Non-epiphyte-lichens like *Cladonia* or *Peltigera* species are mostly confined to the tree base of old trees, where they grow on or in between bryophytes ([43,53]; personal observations). For structural stability, the base of thick-stemmed trees is wide, and the potential habitat available on these trees is therefore larger. Additionally, thick-stemmed trees are usually more structured and offer different microhabitats suitable for non-epiphytes, e.g., large crutches where humus can accumulate. The high richness of bryophyte and lichen species with large diaspores on phenologically old trees might be explained by their low dispersal capacity [24,76]. Generally, large diaspores are rarely transported over large distances, but on old trees the probability of arrival (from remote sources) is higher [8]. The significantly higher number of red-listed lichens on ancient compared to young trees underlines the high conservation value of older trees [74,77,78]

### Environment

Interestingly, we observed opposing altitudinal trends for bryophyte and lichen epiphytes. The richness of epiphyte-bryophytes decreased with increasing altitude, whereas that of lichen-epiphytes increased. Similar results were found on oak trees along an altitudinal gradient in the Mediterranean: the epiphyte richness of lichens increased and that of bryophytes decreased with altitude [79]. It is multifaceted to evaluate the underlying ecological processes of these altitudinal patterns of bryophyte and lichen richness because altitude is a complex factor related to a number of ecological gradients, e.g., decreasing temperature, increasing precipitation or increasing nitrogen deposition [80–82]. The strong positive effect of altitude on the richness of lichens with large diaspores, however, could possibly be related to increased wind speeds at higher altitudes [83], which might favour the dispersal of large diaspores.

Sites close to rivers have often been found to host a large number of epiphytic bryophyte and lichen species, which has been attributed to high humidity [17,18,84]. Our results did not support this relationship. Our study sites are generally characterized by high humidity [36]. Thus, for most species humidity might not be a limiting factor within sites.

Lichens are generally better adapted to higher light levels than bryophytes and, as a consequence, to rapidly changing water relations [9,22,85]. This could explain why a significant effect of radiation was only found for bryophytes in our study. However, epiphyte-bryophytes were unaffected by radiation, indicating that this specialized group of species is well adapted to higher levels of radiation and that this adaptation is necessary to survive on trees [9]. Non-epiphyte-bryophytes were negatively affected by radiation, which is not surprising since many of these non-epiphytes, such as *Brachythecium rutabulum* and *Hylocomium splendens*, usually occur in more shaded habitats (e.g., on the forest floor; [49,54]).

### Isolation

Isolation usually has a negative effect on richness of epiphytes or on the occurrence of single epiphytic species of bryophytes and lichens [10,23,24,86,87]. In our study, isolation exhibited the strongest effect of all predictors on the richness of all lichen species and of epiphyte-lichens but did not significantly affect richness of any bryophyte group. The studies mentioned above found clear relationships between isolation measures and the richness of bryophytes. For example, Boudreault et al. [13] found that distances between burnt and unburnt stands of *Populus tremuloides* significantly affected the richness of epiphytic bryophytes. While the lack of a relationship with isolation measures may be expected for non-epiphytes and for species with small diaspores, which can easily disperse over long distances [88,89], it is more surprising for species with large diaspores. In contrast to other studies (e.g., [13,24]), we sampled only larger trees with a minimum diameter of 36 cm and only a few of the sampled trees were phenologically young. Thus, the trees surveyed in our study were likely considerably older than in other studies, and the time for establishment of epiphytic species was longer. This might partly explain the lack of a relationship between isolation (at spatial scales of hundreds of meters) and the richness of bryophytes. In our study, the strong effect of phenological age and the lack of an effect of isolation measures on the richness of bryophyte species with large diaspores indicate that long-distance dispersal occurs even for this group of species and that establishment is possible if there is enough time available. Further studies are required to assess if these long-distance dispersal events are mediated by the large vegetative propagules or by the rarely produced spores of predominantly asexual species. However, if diaspores are not dispersed by wind but by animals, diaspore size is probably less important.

Most of the lichen species found on the studied sycamore maple trees reproduce sexually and produce spores that are typically small. Dispersal limitation is thus not expected. Indeed, long distance dispersal capacity of lichens (i.e., of the small spores of the mycobiont) is high [90–92], but establishment is generally limited by the availability of the photobiont [21,93,94]. The mycobiont is usually highly selective for the photobiont [95–97], especially in temperate regions [94]. However, little is known about the availability of the photobiont and the dynamics of re-lichenization following sexual reproduction in lichens [33]. Hence, the strong effect of isolation on lichen richness observed here might be due to dispersal limitations of the photobionts (see [98]).

### Drivers of the occurrence of the focal species

The environmental factors we found to be important for the occurrence of *T*. *rudolphiana* confirm what is reported in many floras [30,99,100]: the probability of occurrence of the species increases with altitude, and it typically occurs on north-exposed slopes at sites characterized by high humidity [30,99,100]. The most important variable for the long-term conservation of the species, however, is the negative effect of isolation: as more trees are found within 50 m of a focal tree, the probability that *T*. *rudolphiana* occurs increases. Conservation measures for this species should thus focus on increasing the number of trees in environmentally suitable places. Long distance dispersal of *T*. *rudolphiana* spores by wind is most likely hindered by their adhesive surface, which leads to clumping of the spores [101,102]. How *T*. *rudolphiana* is dispersed is still not well understood. *Tayloria dubyi*, a southern-hemisphere species, was found to be dispersed by flies [103]. During field work for our study, however, we did not observe any indication of insect mediated dispersal of *T*. *rudolphiana*. Furthermore, recruitment from spores might be hindered and a rare event, and dispersal and establishment from vegetative propagules might be more important.

*Lobaria pulmonaria* was only affected by tree characteristics. It is a widespread species and occurs in a variety of ecological conditions [43,104,105]. The ecological conditions of our study sites include the ecological niche of this species, which may have been the reason that we did not find an effect of the environmental variables on probability of occurrence. Previous studies demonstrated clear evidence of dispersal limitation of *L*. *pulmonaria* [105–107]. However, Werth et al. [93] found that vegetative propagules of *L*. *pulmonaria* are also dispersed over larger distances and that stand level ecological conditions probably constrain the occurrence of the species. This is supported by our results, in that no isolation effect was detected for *L*. *pulmonaria*. The unexpected negative effect of DBH on the occurrence probability of *L*. *pulmonaria* might be related to slower rates of bark scaling on thinner (but equally old) trees [9]. *Lobaria pulmonaria* is a late successional species and thus generally depends on microhabitat continuity [108].

According to the classification of During [11], *O*. *rogeri* is a long-lived shuttle species and a weak competitor [109], which was supported by our study. The species was more frequent on younger trees where a large part of the bark is bare. This result also indicates that it is insufficient to consider only old trees (as done by Rosso et al. [110], Fritz et al. [111], Scheidegger and Werth [33]) when addressing conservation measures for endangered epiphyte species.

## Conclusions

The per tree richness of epiphytic bryophytes and lichens is generally influenced by different tree characteristics and by different environmental variables. Furthermore, species with contrasting diaspore sizes, and habitat preferences and different red-list statuses are affected differently by these variables. Tree characteristics related to size and age are usually positively related to richness and the occurrence of red-listed species. However, for some groups of species and for single species, including threatened ones such as *Orthotrichum rogeri*, young or small trees are more important. Thus, for the conservation of high richness and of threatened species in sylvo-pastoral systems, it is crucial to sustain a wide range of tree sizes and ages. Furthermore, our results show that the local abundance of potential diaspore sources (within distances of hundreds of meters) is important for high lichen richness and for the red-listed bryophyte species *Tayloria rudolphiana*. Generally, however, colonization by bryophyte species is not affected from isolation (even for species with large diaspores), and epiphytic bryophyte richness seems to be maintained by (probably rare) long distance dispersal events. The high epiphyte richness and the high number of red-listed species found in sycamore maple wooded pastures highlight the conservation value of these sylvo-pastoral systems. We suggest to established specific conservation policies for sycamore maple wooded pastures in the European Union and in Switzerland.

## Supporting information

S1 Appendix Fig. A. Aerial photo of the study site Grosser Ahornboden (Tyrol, Austria).
Table A. The six study sites.
Fig. B. Sampling design applied to record the per tree richness of bryophytes and lichens on sycamore maple trees.
Table B. Bryophyte and lichen species recorded on 90 sycamore maple trees (*Acer pseudoplatanus*) at six sites in the northern European Alps.
Table C. Initial predictor set considered as fixed effects for the GLMM analyses.
Table D. Correlation table (Spearman’s rho) for the initial predictor set considered for the GLMM analyses.
Table E. Results of the GLMM analyses determining effects on the per tree richness of epiphytic bryophytes and lichens.
Table F. Results of the GLMM analyses determining effects on the occurrence of the three focal species.
(PDF)

S1 Dataset Dataset used for the analyses. (XLSX)
