# Supplementary material for: Epiphytes in wooded pastures: Isolation matters for lichen but not for bryophyte species richness
Source: PLoS One. 2017 Jul 25;12(7):e0182065. doi: 10.1371/journal.pone.0182065 (PMC5526515; doi:10.1371/journal.pone.0182065)
Supplement: S1 Appendix — Fig. A. Aerial photo of the study site Grosser Ahornboden (Tyrol, Austria). Table A. The six study sites. Fig. B. Sampling design applied to record the per tree richness of bryophytes and lichens on sycamore maple trees. Table B. Bryophyte and lichen species recorded on 90 sycamore maple trees (Acer pseudoplatanus) at six sites in the northern European Alps. Table C. Initial predictor set considered as fixed effects for the GLMM analyses. Table D. Correlation table (Spearman’s rho) for the initial predictor set considered for the GLMM analyses. Table E. Results of the GLMM analyses determining effects on the per tree richness of epiphytic bryophytes and lichens. Table F. Results of the GLMM analyses determining effects on the occurrence of the three focal species. (PDF) [file pone.0182065.s001.pdf]

Epiphytes in Wooded Pastures: Isolation Matters for Lichen but not for Bryophyte Species Richness

Thomas Kiebacher, Christine Keller, Christoph Scheidegger & Ariel Bergamini

Corresponding author:
Thomas Kiebacher
Swiss Federal Institute for Forest, Snow and Landscape Research WSL
Zürcherstrasse 111
CH-8903 Birmensdorf
Switzerland
thomas.kiebacher@wsl.ch

Supporting information:

S1 Appendix

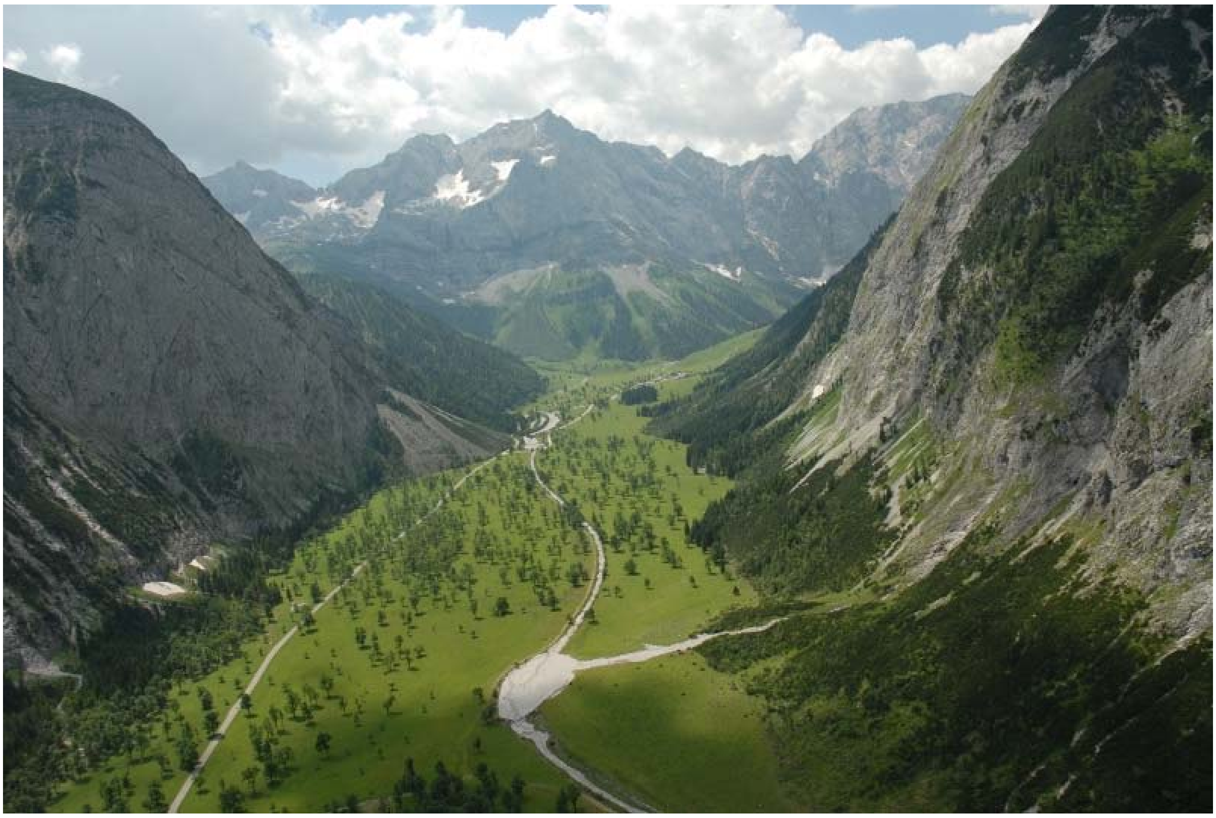

Fig. A. Aerial photo of the study site Grosser Ahornboden (Tyrol, Austria). © G. Haslwanter/Naturpark Karwendel.

Table A. The six study sites. Location, area of the surveyed sycamore maple wooded pastures, number of trees at the sites, number of sampled trees (randomly selected trees + additional trees with occurrence of *Tayloria rudolphiana*) and altitudinal range of the sampled trees.

**Code, Site**	**Location**	**Area [km^2]**	**No. of trees**	**No. of sampled trees**	**Altitudinal range [m a.s.l.]**
**RB**, Reichenbachtal	Schattenhalb and Grindelwald, Bern, Switzerland	2.91	636	16 + 3	1295 - 1529
**MG**, Meniggrund	Diemtigen, Bern, Switzerland	1.69	606	16	1261 - 1444
**GA**, Grosser Ahornboden	Vomp, Tyrol, Austria	1.97	1867	12 + 4	1119 - 1228
**WF**, Wanker Fleck	Halblech, Bavaria, Germany	0.43	93	12	1128 - 1155
**GN**, Gnadenalm	Untertauern, Salzburg, Austria	1.02	148	12 + 3	1048 - 1439
**GT**, Glemmtal	Saalbach-Hinterglemm, Salzburg, Austria	0.26	55	12	1181 - 1394

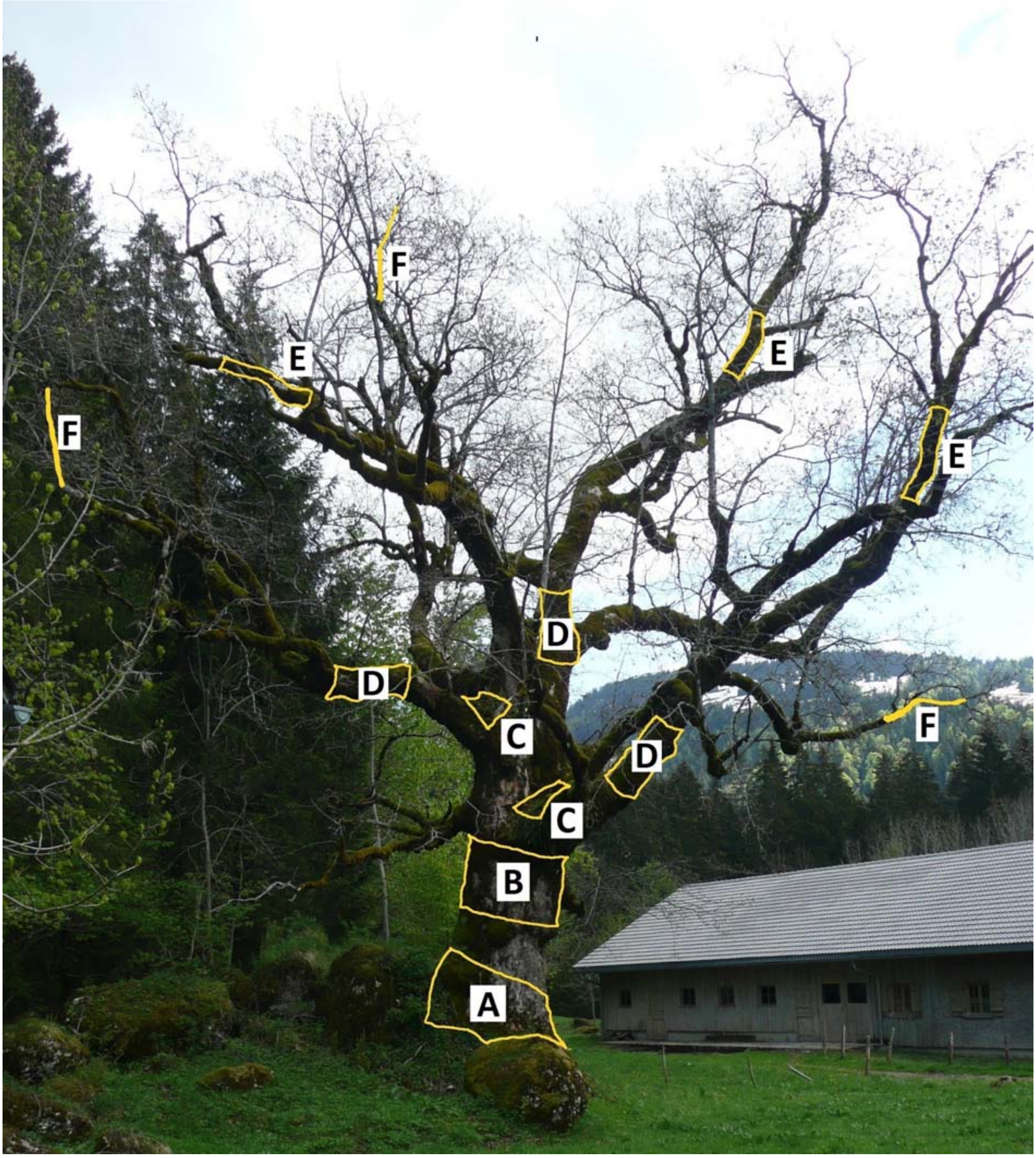

Fig. B. Sampling design applied to record the per tree richness of bryophytes and lichens on sycamore maple trees. A total of 13 plots were placed in the different microhabitats of each focal tree: one plot was placed on the tree base (A), one on the stem below the crown (B), two in the major crutches (C), three on the major branches (D), three on branches of intermediate thickness (E), and three on thin branches in the outer crown (F). Within each plot, the presence of all bryophyte and lichen species was recorded.

Table B. Bryophyte and lichen species recorded on 90 sycamore maple trees (*Acer pseudoplatanus*) at six sites in the northern European Alps. Red-list status (RL) according to Schnyder et al. (2004) and Scheidegger et al. (2002): CR critically endangered, EN endangered, VU vulnerable, NT near threatened, LC least concern, DD data deficiency, NE not evaluated. Species not included in Schnyder et al. (2004) or Scheidegger et al. (2002) are listed as NE*. Red-listed species (CR, EN or VU) are printed in bold. Size of predominant diaspores: l large diaspores, s small diaspores. Habitat preference: E epiphyte, nE non-epiphyte, uc unclassified.

No.	Taxon	RL	Diaspore size	Habitat preference
**Bryophytes**				
1	*Abietinella abietina* (Hedw.) M.Fleisch.	LC	l	nE
2	*Amblystegium serpens* (Hedw.) Schimp.	NE	s	nE
3	*Amblystegium subtile* (Hedw.) Schimp.	LC	s	E
4	*Anomodon attenuatus* (Hedw.) Huebener	LC	l	nE
5	*Anomodon longifolius* (Brid.) Hartm.	LC	l	nE
6	*Anomodon viticulosus* (Hedw.) Hook. & Taylor	LC	l	nE
7	*Antitrichia curtipendula* (Hedw.) Brid.	NE	l	E
8	*Apometzgeria pubescens* (Schrank) Kuwah.	LC	l	nE
9	*Atrichum undulatum* (Hedw.) P.Beauv.	LC	s	nE
10	*Barbilophozia barbata* (Schreb.) Loeske	LC	l	nE
11	*Barbilophozia lycopodioides* (Wallr.) Loeske	LC	l	nE
12	*Barbula unguiculata* Hedw.	LC	s	nE
13	*Bartramia halleriana* Hedw.	LC	s	nE
14	*Blepharostoma trichophyllum* (L.) Dumort. subsp. *trichophyllum*	LC	s	nE
15	*Brachytheciastrum velutinum* (Hedw.) Ignatov & Huttunen	LC	s	nE
16	*Brachythecium albicans* (Hedw.) Schimp.	LC	l	nE
**17**	***Brachythecium campestre* (Müll.Hal.) Schimp.**	**VU**	**s**	**nE**
**18**	***Brachythecium geheebii* Milde**	**CR**	**l**	**nE**
19	*Brachythecium glareosum* (Spruce) Schimp.	LC	l	nE
20	*Brachythecium rivulare* Schimp.	LC	s	nE
21	*Brachythecium rutabulum* (Hedw.) Schimp.	LC	s	nE
22	*Brachythecium salebrosum* (F.Weber & D.Mohr) Schimp., nom. cons.	LC	s	nE
23	*Brachythecium tommasinii* (Boulay) Ignatov & Huttunen	LC	l	nE
24	*Bryoerythrophyllum ferruginascens* (Stirt.) Giacom.	LC	l	nE
25	*Bryoerythrophyllum recurvirostrum* (Hedw.) P.C.Chen	LC	s	nE
26	*Bryum caespiticium* aggr.	NE*	s	nE
27	*Bryum capillare* Hedw.	LC	s	nE
28	*Bryum elegans* Nees	NE*	l	nE
29	*Bryum moravicum* Podp.	LC	l	E
30	*Calliergonella cuspidata* (Hedw.) Loeske	LC	l	nE
31	*Calliergonella lindbergii* (Mitt.) Hedenäs	LC	l	nE
32	*Calypogeia azurea* Stotler & Crotz	LC	l	nE
33	*Campyliadelphus chrysophyllus* (Brid.) R.S.Chopra	LC	l	nE
34	*Campylium stellatum* aggr.	LC	l	nE
35	*Cephalozia* spec. (Dumort.) Dumort.	NE*	uc	nE
36	*Chiloscyphus pallescens* (Hoffm.) Dumort.	LC	s	nE
37	*Cirriphyllum piliferum* (Hedw.) Grout	LC	l	nE
38	*Climacium dendroides* (Hedw.) F.Weber & D.Mohr	LC	l	nE
39	*Conocephalum salebrosum* Szweyk. & al.	NE*	l	nE
40	*Cratoneuron filicinum* (Hedw.) Spruce	LC	l	nE
41	*Ctenidium molluscum* (Hedw.) Mitt.	LC	l	nE
42	*Dichodontium pellucidum* (Hedw.) Schimp.	LC	l	nE
43	*Dicranella varia* (Hedw.) Schimp.	LC	s	nE
44	*Dicranum scoparium* Hedw.	LC	s	nE
45	*Didymodon* cf. *fallax* (Hedw.) R.H.Zander	NE*	uc	nE
46	*Didymodon* cf. *ferrugineus* (Besch.) M.O.Hill	NE*	uc	nE

47	*Ditrichum gracile* (Mitt.) Kuntze	NE*	l	nE
48	*Encalypta streptocarpa* Hedw.	LC	l	nE
49	*Entodon concinnus* (De Not.) Paris	LC	l	nE
50	*Eurhynchium angustirete* (Broth.) T.J.Kop.	LC	l	nE
51	*Fissidens bryoides* aggr.	NE*	s	nE
52	*Fissidens dubius* P.Beauv.	LC	s	nE
53	*Fissidens taxifolius* Hedw.	NE	s	nE
54	*Frullania dilatata* (L.) Dumort.	LC	s	E
55	*Frullania tamarisci* (L.) Dumort.	NT	s	E
56	*Hedwigia ciliata* (Hedw.) P.Beauv.	LC	s	nE
57	*Homalothecium philippeanum* (Spruce) Schimp.	LC	l	nE
58	*Homalothecium sericeum* (Hedw.) Schimp.	LC	l	nE
59	*Homomallium incurvatum* (Brid.) Loeske	LC	s	nE
60	*Hygrohypnum luridum* (Hedw.) Jenn.	LC	s	nE
61	*Hylocomiastrum pyrenaicum* (Spruce) M.Fleisch.	LC	l	nE
62	*Hylocomium splendens* (Hedw.) Schimp.	LC	l	nE
63	*Hypnum andoi* A.J.E.Sm.	LC	s	E
64	*Hypnum cupressiforme* Hedw.	LC	s	nE
65	*Isopterygiopsis muelleriana* (Schimp.) Z.Iwats.	LC	l	nE
66	*Isopterygiopsis pulchella* (Hedw.) Z.Iwats.	LC	s	nE
67	*Isothecium alopecuroides* (Dubois) Isov.	LC	s	E
68	*Jungermannia* spec. L.	NE*	uc	nE
69	*Leiocolea collaris* (Nees) Schljakov	LC	l	nE
70	*Leiocolea heterocolpos* (Hartm.) H.Buch	LC	l	nE
71	*Lejeunea cavifolia* (Ehrh.) Lindb.	LC	s	E
72	*Lescuraea saxicola* (Schimp.) Molendo	LC	l	nE
73	*Leucodon sciuroides* (Hedw.) Schwägr.	LC	l	E
74	*Lophocolea bidentata* (L.) Dumort.	LC	s	nE
75	*Lophocolea heterophylla* (Schrad.) Dumort.	LC	s	nE
76	*Lophocolea minor* Nees	LC	l	nE
77	*Lophozia excisa* (Dicks.) Dumort.	LC	s	nE
78	*Marchantia polymorpha* subsp. *montivagans* Bischl. & Boissel.-Dub.	NE*	l	nE
79	*Metzgeria furcata* (L.) Dumort.	LC	l	E
80	*Metzgeria violacea* (Ach.) Dumort.	LC	l	E
81	*Mnium lycopodioides* Schwägr.	NT	s	nE
82	*Mnium marginatum* (Dicks.) P.Beauv.	LC	s	nE
83	*Mnium spinosum* (Voit) Schwägr.	LC	s	nE
84	*Mnium stellare* Hedw.	LC	l	nE
85	*Mnium thomsonii* Schimp.	LC	s	nE
86	*Neckera complanata* (Hedw.) Huebener	LC	l	E
87	*Orthotrichum affine* Brid.	LC	s	E
88	***Orthotrichum alpestre* Bruch & Schimp.**	**EN**	**s**	**E**
89	*Orthotrichum lyellii* Hook. & Taylor	LC	l	E
90	*Orthotrichum obtusifolium* Brid.	LC	l	E
91	*Orthotrichum pallens* Brid.	LC	s	E
92	*Orthotrichum pumilum* Sw. ex anon.	NE*	s	E
93	***Orthotrichum rogeri* Brid.**	**VU**	**s**	**E**
94	***Orthotrichum scanicum* Gronvall**	**CR**	**s**	**E**
95	*Orthotrichum schimperi* Hammar	NE*	s	E
96	*Orthotrichum speciosum* Nees	LC	s	E
97	***Orthotrichum stellatum* Brid.**	**CR**	**s**	**E**
98	*Orthotrichum stramineum* Brid.	LC	s	E
99	*Orthotrichum striatum* Hedw.	LC	s	E
100	*Oxyrrhynchium hians* (Hedw.) Loeske	LC	l	nE
101	*Oxyrrhynchium schleicheri* (R.Hedw.) Röll	LC	l	nE
102	*Oxystegus tenuirostris* (Hook. & Taylor) A.J.E.Sm.	LC	l	nE

103	*Paraleucobryum sauteri* (Bruch & Schimp.) Loeske	DD	s	E
104	*Pellia* cf. *endiviifolia* (Dicks.) Dumort.	NE*	l	nE
105	*Plagiochila asplenioides* (L. emend. Taylor) Dumort.	LC	l	nE
**106**	***Plagiochila britannica* Paton**	**VU**	**l**	**nE**
107	*Plagiochila porelloides* (Nees) Lindenb.	LC	l	nE
108	*Plagiomnium affine* (Funck) T.J.Kop.	LC	s	nE
109	*Plagiomnium cuspidatum* (Hedw.) T.J.Kop.	LC	s	nE
110	*Plagiomnium elatum* (Bruch & Schimp.) T.J.Kop.	LC	l	nE
111	*Plagiomnium medium* (Bruch & Schimp.) T.J.Kop.	LC	s	nE
112	*Plagiomnium rostratum* (Schrad.) T.J.Kop.	LC	s	nE
113	*Plagiomnium undulatum* (Hedw.) T.J.Kop.	LC	s	nE
114	*Plagiothecium cavifolium* (Brid.) Z.Iwats.	LC	l	nE
115	*Plagiothecium denticulatum* (Hedw.) Schimp.	LC	s	nE
116	*Plagiothecium laetum* Schimp.	LC	s	E
117	*Plagiothecium succulentum* (Wilson) Lindb.	LC	l	nE
118	*Platydictya jungermannioides* (Brid.) H.A.Crum	LC	l	nE
119	*Platygyrium repens* (Brid.) Schimp.	LC	l	E
120	*Pleurozium schreberi* (Brid.) Mitt.	LC	l	nE
121	*Pogonatum urnigerum* (Hedw.) P.Beauv.	LC	s	nE
122	*Pohlia cruda* (Hedw.) Lindb.	LC	l	nE
123	*Pohlia longicolla* (Hedw.) Lindb.	LC	s	nE
124	*Pohlia* cf. *nutans* (Hedw.) Lindb.	NE*	s	nE
125	*Pohlia wahlenbergii* (F.Weber & D.Mohr) A.L.Andrews	LC	l	nE
126	*Polytrichastrum alpinum* (Hedw.) G.L.Sm.	LC	s	nE
127	*Polytrichastrum formosum* (Hedw.) G.L.Sm.	LC	s	nE
128	*Polytrichum commune* Hedw.	LC	s	nE
129	*Polytrichum piliferum* Hedw.	LC	s	nE
130	*Porella platyphylla* (L.) Pfeiff.	LC	l	E
131	*Preissia quadrata* (Scop.) Nees	LC	l	nE
132	*Pseudoleskea incurvata* (Hedw.) Loeske	LC	l	nE
133	*Pseudoleskeella catenulata* (Schrad.) Kindb.	NE	l	nE
134	*Pseudoleskeella nervosa* (Brid.) Nyholm	LC	l	E
135	*Pterigynandrum filiforme* Hedw.	LC	s	E
136	*Ptilidium pulcherrimum* (Weber) Vain.	LC	l	nE
137	*Ptychodium plicatum* (F.Weber & D.Mohr) Schimp.	LC	l	nE
138	*Pylaisia polyantha* (Hedw.) Schimp.	LC	s	E
139	*Racomitrium canescens* (Hedw.) Brid.	LC	l	nE
140	*Radula complanata* (L.) Dumort.	LC	s	E
141	*Rhizomnium punctatum* (Hedw.) T.J.Kop.	LC	s	nE
142	*Rhodobryum roseum* (Hedw.) Limpr.	LC	l	nE
143	*Rhynchostegium murale* (Hedw.) Schimp.	LC	s	nE
144	*Rhytidiadelphus squarrosus* (Hedw.) Warnst.	LC	l	nE
145	*Rhytidiadelphus subpinnatus* (Lindb.) T.J.Kop.	LC	l	nE
146	*Rhytidiadelphus triquetrus* (Hedw.) Warnst.	LC	l	nE
147	*Sanionia uncinata* (Hedw.) Loeske	LC	s	nE
148	*Scapania aequiloba* (Schwägr.) Dumort.	LC	l	nE
149	*Scapania aspera* Bernet & M.Bernet	LC	l	nE
150	*Scapania scandica* (Arnell & H.Buch) Macvicar	LC	l	nE
151	*Schistidium apocarpum* (Hedw.) Bruch & Schimp.	DD	s	nE
152	*Schistidium dupretii* (Thér.) W.A.Weber	NE*	s	nE
153	*Schistidium lancifolium* (Kindb.) H.H.Blom	NE*	s	nE
154	*Schistidium trichodon* var. *nutans* H.H.Blom	NE*	s	nE
155	*Sciuro-Hypnum plumosum* (Hedw.) Ignatov & Huttunen, nom. cons.	LC	s	nE
156	*Sciuro-Hypnum populeum* (Hedw.) Ignatov & Huttunen	LC	s	nE
157	*Sciuro-Hypnum reflexum* (Starke) Ignatov & Huttunen	LC	s	nE
158	*Sciuro-Hypnum starkei* (Brid.) Ignatov & Huttunen	LC	s	nE

159	*Syntrichia norvegica* F.Weber	LC	s	nE
160	*Syntrichia ruralis* (Hedw.) F.Weber & D.Mohr	LC	s	E
161	*Syntrichia virescens* (De Not.) Ochyra	LC	l	E
162	***Tayloria rudolphiana* (Garov.) Bruch & Schimp.**	**VU**	**s**	**E**
163	*Tayloria serrata* (Hedw.) Bruch & Schimp.	LC	l	nE
164	*Thamnobryum neckeroides* (Hook.) E.Lawton	NE*	l	nE
165	*Thuidium assimile* (Mitt.) A.Jaeger	LC	l	nE
166	*Thuidium delicatulum* (Hedw.) Schimp.	LC	l	nE
167	*Tortella bambergeri* (Schimp.) Broth.	LC	l	nE
168	*Tortella tortuosa* (Hedw.) Limpr.	LC	l	nE
169	*Tortula subulata* Hedw.	LC	s	nE
170	*Trichodon cylindricus* (Hedw.) Schimp.	LC	l	nE
171	*Ulota bruchii* Brid.	LC	s	E
172	***Ulota coarctata* (P.Beauv.) Hammar**	**CR**	**s**	**E**
173	*Ulota crispa* (Hedw.) Brid.	LC	s	E
174	*Weissia controversa* Hedw.	LC	s	nE
175	*Zygodon dentatus* (Limpr.) Kartt.	NT	l	E
176	***Zygodon rupestris* Lorentz**	**VU**	**l**	**E**

Lichens

1	*Agonimia tristicula* (Nyl.) Zahlbr.	LC	l	E
2	***Anaptychia ciliaris* (L.) Körb.**	**VU**	**s**	**E**
3	*Arthonia atra* (Pers.) A. Schneid.	LC	s	E
4	*Arthonia didyma* Körb.	LC	s	E
5	*Arthonia punctiformis* Ach.	NE*	s	E
6	*Arthonia radiata* (Pers.) Ach.	LC	s	E
7	*Arthopyrenia* cf. *carneobrunneola* Coppins	NE*	s	E
8	*Arthopyrenia* cf. *salicis* A. Massal.	NE*	s	E
9	*Bacidia arceutina* (Ach.) Arnold	LC	l	E
10	*Bacidia arnoldiana* aggr.	NE*	s	uc
11	*Bacidia beckhausii* Körb.	NT	l	E
12	***Bacidia circumspecta* (Vain.) Malme**	**EN**	**l**	**E**
13	*Bacidia rubella* (Hoffm.) A. Massal.	LC	l	E
14	*Bacidia subincompta* (Nyl.) Arnold	LC	l	E
15	*Bacidia* cf. *vermifera* (Nyl.) Th. Fr.	NE*	l	E
16	*Bacidia* cf. *viridifarinosa* Coppins & P. James	NE*	s	uc
17	*Biatora chrysantha* (Zahlbr.) Printzen	LC	s	E
18	*Biatora efflorescens* (Hedl.) Räsänen	LC	s	E
19	*Biatora flavopunctata* (Tønsberg) Hinter. & Printzen	LC	s	E
20	*Biatora helvola* Hellb.	NT	s	E
21	*Biatoridium monasteriense* Körb.	LC	s	E
22	*Bilimbia sabuletorum* (Schreb.) Arnold	LC	s	nE
23	***Bryoria bicolor* (Ehrh.) Brodo & D. Hawksw.**	**VU**	**l**	**E**
24	*Bryoria capillaris* (Ach.) Brodo & D. Hawksw.	NT	l	E
25	*Bryoria fuscescens* (Gyeln.) Brodo & D. Hawksw.	LC	l	E
26	*Bryoria implexa* (Hoffm.) Brodo & D. Hawksw.	NT	l	E
27	***Buellia erubescens* Arnold**	**VU**	**s**	**E**
28	*Buellia griseovirens* (Sm.) Almb.	LC	s	E
29	*Buellia punctata* (Hoffm.) A. Massal.	LC	s	E
30	*Calicium* cf. *glaucellum* Ach.	NE*	s	E
31	*Callopisma* cf. *asserigenum* J. Lahm	NE*	s	E
32	***Caloplaca alnetorum* Giralt & al.**	**VU**	**s**	**E**
33	*Caloplaca alstrupii* Søchting	NE*	s	E
34	*Caloplaca cerina* (Hedw.) Th. Fr.	LC	s	E
35	*Caloplaca cerinella* (Nyl.) Flagey	NT	s	E
36	*Caloplaca cerinelloides* (Erichsen) Poelt	NT	s	E

37	*Caloplaca chlorina* (Flot.) H. Olivier	LC	s	nE
38	*Caloplaca herbidella* (Hue) H. Magn.	LC	s	E
39	*Caloplaca hungarica* H. Magn.	NE*	s	E
40	*Caloplaca* cf. *obscurella* (Körb.) Th. Fr.	NE*	s	E
41	*Candelaria concolor* (Dicks.) Stein	LC	l	E
42	*Candelariella efflorescens* aggr.	NE*	s	E
43	*Candelariella reflexa* (Nyl.) Lettau	LC	s	E
44	*Candelariella vitellina* (Hoffm.) Müll. Arg.	LC	l	nE
45	*Candelariella xanthostigma* (Ach.) Lettau	LC	l	E
46	*Catillaria nigroclavata* (Nyl.) Schuler	LC	s	E
47	***Cetraria sepincola* (Ehrh.) Ach.**	**EN**	**s**	**E**
48	*Cetrelia olivetorum* (Nyl.) W. L. Culb. & C. F. Culb.	NT	s	E
49	*Chrysothrix candelaris* (L.) J. R. Laundon	LC	s	E
50	*Cladonia chlorophaea* (Sommerf.) Spreng.	NE*	l	nE
51	*Cladonia coniocraea* (Flörke) Spreng.	LC	s	E
52	*Cladonia fimbriata* (L.) Fr.	LC	s	nE
53	*Cladonia pocillum* (Ach.) Grognot	LC	l	nE
54	*Cladonia pyxidata* (L.) Hoffm.	LC	l	nE
55	***Collema fasciculare* (L.) F. H. Wigg.**	**EN**	**s**	**E**
56	*Collema flaccidum* (Ach.) Ach.	LC	l	E
57	***Collema fragrans* (Sm.) Ach.**	**CR**	**s**	**E**
58	*Collema* cf. *ligerinum*(Hy) Harm.	NE*	s	E
59	***Collema nigrescens* aggr.**	**VU**	**l**	**E**
60	*Diploschistes muscorum* (Scop.) R. Sant.	LC	l	nE
61	***Diplotomma alboatrum* (Hoffm.) Flot.**	**EN**	**s**	**E**
62	*Evernia divaricata* (L.) Ach.	NT	l	E
63	*Evernia prunastri* (L.) Ach.	LC	s	E
64	***Fellhanera subtilis* (Vezda) Diederich & Sérus.**	**VU**	**s**	**E**
65	*Frutidella pullata* (Norman) Schmull	LC	s	E
66	*Heterodermia* cf. *japonica* (M. Satô) Swinscow & Krog	NE*	s	E
67	***Heterodermia speciosa* (Wulfen) Trevis.**	**CR**	**s**	**E**
68	*Hypogymnia austerodes* (Nyl.) Räsänen	LC	s	E
69	*Hypogymnia bitteri* (Lynge) Ahti	LC	s	E
70	*Hypogymnia farinacea* Zopf	LC	s	E
71	*Hypogymnia physodes* (L.) Nyl.	LC	s	E
72	*Hypogymnia tubulosa* (Schaer.) Hav.	LC	s	E
73	***Hypogymnia vittata* (Ach.) Parrique**	**VU**	**s**	**E**
74	*Hypotrachyna afrorevoluta* (Krog & Swinscow) Krog &	NE*	s	E
75	*Lecania cyrtella* (Ach.) Th. Fr.	LC	s	E
76	*Lecania hyalina* (Fr.) R. Sant.	NT	s	E
77	*Lecanora albella* (Pers.) Ach.	NT	s	E
78	*Lecanora albescens* (Hoffm.) Branth & Rostr.	NE*	s	nE
79	*Lecanora allophana* f. *allophana* Nyl.	NT	s	E
80	*Lecanora allophana* f. *sorediata* Vain.	NE*	s	E
81	*Lecanora argentata* (Ach.) Malme	LC	s	E
82	*Lecanora barkmaniana* Aptroot & Herk	LC	s	E
83	*Lecanora carpinea* (L.) Vain.	LC	s	E
84	*Lecanora chlarotera* Nyl.	LC	s	E
85	*Lecanora circumborealis* Brodo & Vitik.	LC	s	E
86	*Lecanora expersa* Nyl.	LC	s	E
87	*Lecanora hagenii* (Ach.) Ach.	NE*	s	E
88	*Lecanora intumescens* (Rebent.) Rabenh.	NT	s	E
89	*Lecanora leptyrodes* (Nyl.) Degel.	NT	s	E
90	*Lecanora persimilis* (Th. Fr.) Nyl.	LC	s	E
91	*Lecanora praesistens* Nyl.	NT	s	E
92	*Lecanora pulicaris* (Pers.) Ach.	LC	s	E

93	*Lecanora strobilina* (Spreng.) Kieff.	NE*	s	E
94	*Lecanora subcarpinea* Szatala	NT	s	E
95	*Lecanora symmicta* (Ach.) Ach.	LC	s	E
96	*Lecanora umbrina* (Ach.) A. Massal.	NE*	s	E
97	*Lecidea* cf. *albohyalina* (Nyl.) Th.Fr.	NE*	s	E
98	*Lecidea nylanderi* (Anzi) Th. Fr.	LC	s	E
99	*Lecidella elaeochroma* var. *elaeochroma* (Ach.) M. Choisy	LC	s	E
100	*Lecidella flavosorediata* (Vezda) Hertel & Leuckert	LC	s	E
101	*Lepraria coriensis* (Hue) Sipman	NE*	s	nE
102	*Lepraria eburnea* J. R. Laundon	LC	s	E
103	*Lepraria elobata* Tønsberg	LC	s	E
104	*Lepraria lobificans* Nyl.	LC	s	E
105	*Lepraria membranacea* (Dicks.) Vain.	NE*	s	nE
106	*Lepraria rigidula* (B. de Lesd.) Tønsberg	LC	s	E
107	*Lepraria vouauxii* (Hue) R. C. Harris	LC	s	E
108	*Leptogium lichenoides* (L.) Zahlbr.	LC	l	nE
109	*Leptogium saturninum* (Dicks.) Nyl.	NT	s	E
**110**	***Leptogium teretiusculum* (Wallr.) Arnold**	**EN**	**l**	**E**
**111**	***Lobaria amplissima* (Scop.) Forssell**	**EN**	**l**	**E**
**112**	***Lobaria pulmonaria* (L.) Hoffm.**	**VU**	**l**	**E**
113	*Loxospora elatina* (Ach.) A. Massal.	LC	s	E
114	*Melanelixia glabra* (Schaer.) O. Blanco & al.	NT	s	E
115	*Melanelixia glabratula* (Lamy) Sandler & Arup	LC	s	E
116	*Melanelixia subargentifera* (Nyl.) O. Blanco & al.	LC	s	E
117	*Melanelixia subaurifera* (Nyl.) O. Blanco & al.	LC	s	E
118	*Melanohalea elegantula* (Zahlbr.) O. Blanco & al.	NT	s	E
119	*Melanohalea exasperata* (De Not.) O. Blanco & al.	NT	s	E
120	*Melanohalea exasperatula* (Nyl.) O. Blanco & al.	LC	l	E
**121**	***Menegazzia terebrata* (Hoffm.) A. Massal.**	**VU**	**s**	**E**
122	*Micarea prasina* s.lat.	LC	s	E
123	*Mycobilimbia epixanthoides* (Nyl.) Hafellner & Türk	LC	s	E
124	*Mycobilimbia tetramera* (De Not.) Hafellner & Türk	NE*	s	E
**125**	***Mycoblastus affinis* (Schaer.) T. Schauer**	**VU**	**l**	**E**
126	*Nephroma bellum* (Spreng.) Tuck.	NT	s	E
127	*Nephroma parile* (Ach.) Ach.	NT	s	E
**128**	***Nephroma resupinatum* (L.) Ach.**	**VU**	**s**	**E**
**129**	***Nephromopsis laureri* (Kremp.) Kurok.**	**VU**	**s**	**E**
130	*Normandina pulchella* (Borrer) Nyl.	LC	s	E
131	*Ochrolechia alboflavescens* (Wulfen) Zahlbr.	LC	s	E
132	*Ochrolechia androgyna* (Hoffm.) Arnold	LC	s	E
133	*Ochrolechia arborea* (Kreyer) Almb.	NT	s	E
**134**	***Ochrolechia pallescens* (L.) A. Massal.**	**EN**	**l**	**E**
**135**	***Ochrolechia szatalaensis* Verseghy**	**VU**	**l**	**E**
136	*Ochrolechia* cf. *turneri* (Sm.) Hasselrot	NE*	s	E
137	*Opegrapha rufescens* Pers.	LC	s	E
138	*Opegrapha varia* Pers.	NE*	s	E
139	*Oxneria huculia* S.Y. Kondr	NE*	s	E
**140**	***Pachyphiale fagicola* (Hepp) Zwackh**	**VU**	**s**	**E**
**141**	***Pannaria conoplea* (Ach.) Bory**	**EN**	**s**	**E**
142	*Parmelia ernstiae* Feuerer & A. Thell	NE*	s	E
143	*Parmelia saxatilis* (L.) Ach.	NE*	s	E
144	*Parmelia serrana* A. Crespo, M.C. Molina & D. Hawksw.	NE*	s	E
145	*Parmelia submontana* Hale	LC	s	E
146	*Parmelia sulcata* Taylor	LC	s	E
147	*Parmeliella triptophylla* (Ach.) Müll. Arg.	NT	l	E
148	*Parmelina carporrhizans* (Taylor) Poelt & Vezda	NE*	s	E

149	*Parmelina pastillifera* (Harm.) Hale	NT	l	E
150	*Parmelina quercina* (Willd.) Hale	NE*	s	E
151	*Parmelina tiliacea* (Hoffm.) Hale	LC	s	E
152	***Parmotrema arnoldii* (Du Rietz) Hale**	**VU**	**s**	**E**
153	*Peltigera canina* (L.) Willd.	LC	l	nE
154	*Peltigera collina* (Ach.) Schrad.	NT	s	E
155	*Peltigera didactyla* (With.) J. R. Laundon	LC	s	nE
156	*Peltigera elisabethae* Gyeln.	LC	s	nE
157	*Peltigera horizontalis* (Huds.) Baumg.	NT	s	nE
158	*Peltigera membranacea* (Ach.) Nyl.	NT	l	nE
159	*Peltigera polydactylon* (Neck.) Hoffm.	LC	l	nE
160	*Peltigera praetextata* (Sommerf.) Zopf	LC	l	nE
161	*Pertusaria albescens* (Huds.) M. Choisy & Werner	LC	s	E
162	*Pertusaria amara* (Ach.) Nyl.	LC	s	E
163	***Pertusaria coccodes* (Ach.) Nyl.**	**VU**	**s**	**E**
164	***Pertusaria coronata* (Ach.) Th. Fr.**	**VU**	**s**	**E**
165	***Pertusaria hemisphaerica* (Flörke) Erichsen**	**EN**	**s**	**E**
166	*Pertusaria leioplaca* DC.	LC	l	E
167	***Pertusaria multipuncta* (Turner) Nyl.**	**EN**	**l**	**E**
168	*Pertusaria pupillaris* (Nyl.) Th. Fr.	NT	s	nE
169	*Phaeophyscia ciliata* (Hoffm.) Moberg	NT	s	E
170	*Phaeophyscia endophoenicea* (Harm.) Moberg	LC	s	E
171	*Phaeophyscia hirsuta* (Mereschk.) Essl.	NT	s	E
172	*Phaeophyscia orbicularis* (Neck.) Moberg	LC	s	E
173	*Phlyctis argena* (Spreng.) Flot.	LC	s	E
174	*Physcia adscendens* (Fr.) H. Olivier	LC	s	E
175	*Physcia aipolia* (Humb.) Fürnr.	LC	s	E
176	*Physcia caesia* (Hoffm.) Fürnr.	NE*	s	nE
177	*Physcia dubia* (Hoffm.) Lettau	NE*	s	nE
178	*Physcia stellaris* (L.) Nyl.	LC	s	E
179	*Physcia tenella* (Scop.) DC.	LC	s	E
180	*Physconia distorta* (With.) J. R. Laundon	LC	s	E
181	*Physconia enteroxantha* (Nyl.) Poelt	NT	s	E
182	*Physconia grisea* (Lam.) Poelt	NT	s	E
183	*Physconia perisidiosa* (Erichsen) Moberg	NT	s	E
184	*Placynthiella icmalea* (Ach.) Coppins & P. James	LC	s	nE
185	*Placynthiella uliginosa* (Schrad.) Coppins & P. Jam	LC	s	nE
186	*Platismatia glauca* (L.) W. L. Culb. & C. F. Culb.	LC	s	E
187	*Porina aenea* (Wallr.) Zahlbr.	LC	s	E
188	*Pseudevernia furfuracea* (L.) Zopf	LC	s	E
189	*Punctelia subrudecta* (Nyl.) Krog	NE*	s	E
190	*Ramalina calicaris* (L.) Fr.	NE*	s	E
191	*Ramalina farinacea* (L.) Ach.	LC	s	E
192	***Ramalina fastigiata* (Pers.) Ach.**	**VU**	**s**	**E**
193	*Ramalina fraxinea* (L.) Ach.	NT	s	E
194	***Ramalina obtusata* (Arnold) Bitter**	**VU**	**s**	**E**
195	***Ramalina panizzei* De Not.**	**EN**	**s**	**E**
196	*Ramalina pollinaria* (Westr.) Ach.	NT	s	E
197	***Ramalina roesleri* (Schaer.) Hue**	**EN**	**s**	**E**
198	***Ramalina thrausta* (Ach.) Nyl.**	**EN**	**s**	**E**
199	*Rinodina archaea* (Ach.) Arnold	LC	s	nE
200	*Rinodina capensis* Hampe	NT	s	E
201	***Rinodina conradii* Körb.**	**VU**	**s**	**E**
202	*Rinodina* cf. *degeliana* Coppins	NE*	s	E
203	*Rinodina exigua* (Ach.) Gray	NT	s	E
204	*Rinodina griseosoralifera* Coppins	NT	s	E

205	*Rinodina septentrionalis* Malme	LC	s	E
206	*Rinodina sophodes* (Ach.) A. Massal.	NT	s	E
**207**	***Sclerophora pallida* (Pers.) Y. J. Yao & Spooner**	**VU**	**s**	**E**
208	*Scoliciosporum chlorococcum* (Stenh.) Vezda	LC	s	E
209	*Scoliciosporum sarothamni* (Vain.) Vezda	LC	s	E
210	*Scoliciosporum umbrinum* (Ach.) Arnold	LC	s	nE
211	*Strigula stigmatella* (Ach.) R. C. Harris	LC	s	E
**212**	***Thelenella modesta* (Nyl.) Nyl.**	**CR**	**s**	**E**
**213**	***Trapelia corticola* Coppins & P. James**	**VU**	**s**	**E**
214	*Trapeliopsis flexuosa* (Fr.) Coppins & P. James	LC	s	nE
215	*Trapeliopsis* cf. *gelatinosa*_(Flörke) Coppins & P. Jame	NE*	s	nE
216	*Tuckermanopsis chlorophylla* (Willd.) Hale	LC	s	E
217	*Usnea barbata* (L.) F. H. Wigg.	NE*	s	E
218	*Usnea cavernosa* Tuck.	NT	l	E
219	*Usnea dasypoga* (Ach.) Nyl.	NT	s	E
**220**	***Usnea florida* (L.) F. H. Wigg.**	**EN**	**s**	**E**
**221**	***Usnea glabrescens* var. *fulvoreagens* Räsänen**	**VU**	**s**	**E**
**222**	***Usnea intermedia* (A. Massal.) Jatta**	**VU**	**s**	**E**
223	*Usnea lapponica* Vain.	LC	s	E
224	*Usnea subfloridana* Stirt.	LC	s	E
225	*Usnea substerilis* Motyka	LC	s	E
226	*Violella fucata* (Stirt.) T. Sprib.	LC	s	E
227	*Vulpicida pinastri* (Scop.) J.-E. Mattsson & M. J.	LC	s	E
228	*Xanthoria candelaria* (L.) Th. Fr.	LC	s	E
229	*Xanthoria fulva* (Hoffm.) Poelt & Petut.	NT	s	E
230	*Xanthoria parietina* (L.) Th. Fr.	LC	s	E
231	*Xanthoria polycarpa* (Hoffm.) Rieber	LC	s	E
232	*Xanthoria ulophyllodes* Räsänen	NT	s	E

References

Scheidegger, C., P. Clerc, M. Dietrich, M. Frei, U. Groner, C. Keller, I. Roth, S. Stofer, and M. Vust. 2002. Rote Liste der gefährdeten baum- und erdbewohnenden Flechten der Schweiz. WSL, CJB, BUWAL, Bern.

Schnyder, N., A. Bergamini, H. Hofmann, N. Müller, C. Schubiger-Bossard, and E. Urmi. 2004. Rote Liste der gefährdeten Moose der Schweiz. BUWAL-Reihe: Vollzug Umwelt. BUWAL, FUB & NISM, Bern.

Table C. Initial predictor set considered as fixed effects for the GLMM analyses.

Predictor	Description	Transformation
Tree characteristics		
**DBH**	Tree diameter at breast height (1.3 m above ground)	
**Tree height**	Vertical height of the tree	
**Crown volume**	Volume of the crown	
**Phenological age**	Three-level factor: 1 young, 2 mature, 3 ancient	
**Sampled area per tree**	Sum of the area of the 13 plots	
Environmental variables		
**Altitude**	Altitude above sea level	
**Precipitation**	Mean annual precipitation, derived from a 100 m grid interpolated with the algorithm following Zimmermann and Roberts (2001) from the 1950-2000 means of the WorldClim data (Hijmans et al. 2005)	
**Distance to river**	Minimum Euclidian distance to nearest river	Natural logarithm
**Radiation**	Annual global potential shortwave radiation (algorithm following Kumar et al. 1997)	
Isolation measures		
**Minimum distance**	Projected Euclidian distance (derived from orthophotos) to the nearest sycamore maple tree with DBH ≥ 36 cm	
**No.trees50m**	Number of neighbouring trees within a radius of 200 m	Square root
**Pot.habitat50m**	Potential habitat amount within a radius of 50 m: Σ $DBH_i^{0.6}$ of trees with DBH ≥ 36 cm within a radius of 50 m (sensu Moilanen and Nieminen 2002; they used Σ $Area_i^{0.3}$; we used 0.6 instead of 0.3 as the exponent because DBH is linear)	Square root
**No.trees200m**	Number of neighbouring trees within a radius of 200 m	Square root
**Si1**	Connectivity index sensu Hanski (1999) with modifications according to Löbel et al. (2009) with α set to 1 and γ to 0.3 (Moilanen and Nieminen 2002) for trees with DBH ≥ 36 cm within a radius of 50 m of the focal tree.	
**Si2**	Connectivity index sensu Hanski (1999) with modifications according to Löbel et al. (2009) with α set to 1 and γ to 0.3 (Moilanen and Nieminen 2002) trees within a radius of 200 m and DBH set to 1.	

References

Hanski, I. 1999. Habitat connectivity, habitat continuity, and metapopulation in dynamic landscapes.

Hijmans, R. J., S. E. Cameron, J. L. Parra, P. G. Jones, and A. Jarvis. 2005. Very high resolution interpolated climate surfaces for global land areas. International Journal of Climatology 25:1965–1978.

Kumar, L., A. K. Skidmore, and E. Knowles. 1997. Modelling topographic variation in solar radiation in a GIS environment. International Journal of Geographical Information Science 11:475–497.

Löbel, S., T. Snäll, and H. Rydin. 2009. Mating system, reproduction mode and diaspore size affect metacommunity diversity. Journal of Ecology 97:176–185.

Moilanen, A., and M. Nieminen. 2002. Simple connectivity measure in spatial ecology. Ecology 83:1131–1145.

Zimmermann, N. E., and D. W. Roberts. 2001. Final report of the MLP climate and biophysical mapping project. https://www.wsl.ch/staff/niklaus.zimmermann/mlp/mlp_report.pdf.

Table D. Correlation table (Spearman's rho) for the initial predictor set considered for the GLMM analyses. In bold: |Spearman's rho| > 0.7. Height: tree height, DBH: diameter at breast height, CrVol: crown volume, Area: sampled area, PhAge: phenological age (ordinal scale), Alt: altitude, DistRiv: distance to nearest river, Rad: radiation, Prec: precipitation, MinDist: distance to nearest tree, NoTr50: number of trees within a radius of 50 m (square root transformed), NoTr200: number of trees within a radius of 200 m (square root transformed), PotHab50: potential habitat amount within a radius of 50 m, Si1: modified connectivity index sensu Hanski (1999) for trees within a radius of 50 m, Si2: modified connectivity index sensu Hanski (1999) for trees within a radius of 200 m.

	Height	DBH	CrVol	Area	PhAge	Alt	DistRiv	Rad	Prec	MinDist	NoTr50	NoTr200	PotHab50	Si1
**Height**														
**DBH**	0.33													
**CrVol**	**0.74**	0.55												
**Area**	0.38	**0.85**	0.62											
**PhAge**	0.35	0.66	0.33	0.60										
**Alt**	0.04	0.31	0.40	0.41	0.10									
**DistRiv**	-0.12	-0.05	-0.20	-0.08	0.08	-0.28								
**Rad**	0.09	0.02	0.17	0.09	-0.08	0.22	-0.16							
**Prec**	0.02	0.13	0.28	0.23	-0.06	**0.76**	-0.32	0.29						
**MinDist**	-0.10	0.31	0.06	0.35	0.11	0.24	-0.09	-0.03	0.18					
**NoTr50**	0.19	-0.27	0.00	-0.30	-0.07	-0.18	0.11	0.04	-0.20	**-0.74**				
**NoTr200**	0.21	-0.19	0.11	-0.24	-0.01	-0.12	0.18	0.11	-0.23	-0.47	0.69			
**PotHab50**	0.24	-0.21	0.05	-0.24	-0.02	-0.16	0.10	0.06	-0.20	**-0.74**	**0.99**	0.69		
**Si1**	0.19	-0.23	-0.02	-0.28	-0.02	-0.21	0.07	0.04	-0.22	**-0.93**	**0.87**	0.58	**0.88**	
**Si2**	0.14	-0.33	-0.05	-0.37	-0.12	-0.22	0.10	0.07	-0.20	**-0.93**	**0.89**	0.66	**0.88**	**0.96**

References

Hanski, I. 1999. Habitat connectivity, habitat continuity, and metapopulation in dynamic landscapes.

Table E. Results of the GLMM analyses determining effects on the per tree richness of epiphytic bryophytes and lichens. Random terms of the models are given. For fixed effects (tree characteristics, environmental variables and isolation measures) standardized coefficient estimates, relative variable importance (RVI), unconditional standard error (SE), z-value, *p*-value and 95% confidence intervals (CI) after conditional model averaging are shown. Ad.trees: additional trees; ol: observational level; M: number of models in the candidate model set (Δ AICc < 2) considered for averaging estimates; K: number of predictors in the candidate model set; DBH: diameter at breast height; Phen.age: phenological age (1 = young trees, 2 = mature trees, 3 = ancient trees; we used the first level of the factor as the baseline for effects of phenological age); Pot.habitat50m: potential habitat amount within radius of 50 m; No.trees200m: number of trees within radius of 200 m. # $p < 0.1$, * $p < 0.05$, ** $p < 0.01$, *** $p < 0.001$.

Bryophytes

Species group	Random term	M	K	Fixed effect	RVI	Estimate	SE	z-value	p		2.5% CI	97.5% CI
**All species**	(1|Site/Ad.trees)	4	5									
				Crown volume	0.81	0.096	0.048	1.984	0.047	*	0.001	0.191
				Altitude	0.72	0.116	0.067	1.712	0.087	#	-0.017	0.250
				Phen.age2	1.00	0.199	0.062	3.164	0.002	**	0.076	0.321
				Phen.age3		0.280	0.088	3.146	0.002	**	0.105	0.454
				Radiation	1.00	-0.103	0.045	2.253	0.024	*	-0.192	-0.013
				Distance to river	0.17	-0.052	0.054	0.943	0.346		-0.159	0.056
**Red-listed**	(1|Site/Ad.trees)	12	5									
				DBH	0.37	0.245	0.178	1.353	0.176		-0.110	0.599
				Distance to river	0.82	0.430	0.213	1.993	0.046	*	0.007	0.853
				No.trees200m	0.29	-0.241	0.200	1.186	0.236		-0.639	0.157
				Pot.habitat50m	0.28	-0.233	0.202	1.141	0.254		-0.634	0.167
				Crown volume	0.15	0.134	0.193	0.684	0.494		-0.250	0.519
**Not red-listed**	(1|Site/Ad.trees)	7	6									
				Crown volume	0.74	0.090	0.049	1.815	0.069	#	-0.007	0.187
				Phen.age2	1.00	0.218	0.064	3.360	0.001	***	0.091	0.345
				Phen.age3		0.306	0.091	3.326	0.001	***	0.126	0.486
				Radiation	1.00	-0.113	0.046	2.414	0.016	*	-0.205	-0.021
				Altitude	0.41	0.094	0.068	1.368	0.171		-0.041	0.230
				Distance to river	0.23	-0.061	0.055	1.097	0.273		-0.170	0.048
				No.trees200m	0.09	-0.044	0.065	0.669	0.504		-0.174	0.085
**Small diaspores**	(1|Site/Ad.trees)	5	4									
				Crown volume	1.00	0.145	0.061	2.356	0.019	*	0.024	0.265
				No.trees200m	0.60	-0.119	0.073	1.602	0.109		-0.265	0.027
				DBH	0.30	0.062	0.063	0.964	0.335		-0.064	0.188
				Radiation	0.12	-0.032	0.055	0.580	0.562		-0.142	0.077
**Large diaspores**	(1|Site/Ad.trees)	4	5									
				Phen.age2	1.00	0.454	0.108	4.127	0.000	***	0.238	0.669
				Phen.age3		0.622	0.137	4.473	0.000	***	0.350	0.895
				Radiation	1.00	-0.197	0.072	2.711	0.007	**	-0.339	-0.055
				Altitude	0.24	0.124	0.106	1.145	0.252		-0.088	0.335
				Distance to river	0.21	-0.090	0.085	1.048	0.295		-0.260	0.079
				Crown volume	0.16	0.057	0.076	0.737	0.461		-0.094	0.208
**Non-epiphytes**	(1|Site/Ad.trees) + (1|ol)	7	5									
				Altitude	0.46	0.276	0.180	1.515	0.130		-0.081	0.634
				Phen.age2	1.00	0.520	0.168	3.059	0.002	**	0.187	0.854
				Phen.age3		0.809	0.221	3.605	0.000	***	0.369	1.248
				Radiation	0.77	-0.232	0.120	1.910	0.056	#	-0.470	0.006
				Distance to river	0.20	-0.124	0.139	0.876	0.381		-0.400	0.153
				Pot.habitat50m	0.10	-0.143	0.147	0.959	0.338		-0.436	0.150
**Epiphytes**	(1|Site/Ad.trees)	6	5									
				Altitude	0.86	-0.154	0.072	2.114	0.035	*	-0.298	-0.011
				DBH	0.20	0.073	0.055	1.303	0.193		-0.037	0.184
				Crown volume	0.19	0.073	0.057	1.269	0.205		-0.040	0.186
				No.trees200m	0.11	-0.048	0.063	0.749	0.454		-0.173	0.077
				Distance to river	0.10	0.038	0.062	0.610	0.542		-0.085	0.162
**- Red-listed**	(1|Site/Ad.trees)	8	6									
				Distance to river	0.83	0.448	0.201	2.199	0.028	*	0.049	0.848
				No.trees200m	0.18	-0.254	0.177	1.416	0.157		-0.606	0.098
				Pot.habitat50m	0.13	-0.212	0.173	1.203	0.229		-0.556	0.133
				DBH	0.22	0.219	0.174	1.245	0.213		-0.126	0.565
				Crown volume	0.09	0.145	0.188	0.760	0.447		-0.229	0.520
				Altitude	0.08	0.128	0.227	0.555	0.579		-0.324	0.580
**- Not red-listed**	(1|Site/Ad.trees)	4	4									
				Altitude	1.00	-0.185	0.065	2.794	0.005	**	-0.314	-0.055
				Crown volume	0.24	0.067	0.059	1.125	0.261		-0.050	0.184

				DBH	0.21	0.058	0.058	0.991	0.322	-0.057	0.174
				No.trees200m	0.16	-0.044	0.060	0.713	0.476	-0.164	0.076
**- Small diaspores**	(1|Site/Ad.trees)	4	3								
				Altitude	0.27	-0.119	0.090	1.312	0.189	-0.298	0.059
				Distance to river	0.20	0.076	0.079	0.953	0.340	-0.080	0.232
				Crown volume	0.15	0.043	0.072	0.589	0.556	-0.101	0.187
**- Large diaspores**	(1|Site/Ad.trees)	11	7								
				DBH	0.37	0.151	0.090	1.657	0.098 #	-0.028	0.329
				Altitude	1.00	-0.226	0.095	2.335	0.020 *	-0.415	-0.036
				Phen.age2	0.35	0.268	0.131	2.011	0.044 *	0.007	0.529
				Phen.age3		0.317	0.172	1.821	0.069 #	-0.024	0.659
				Crown volume	0.09	0.120	0.091	1.302	0.193	-0.061	0.300
				No.trees200m	0.21	-0.090	0.092	0.963	0.336	-0.272	0.093
				Radiation	0.14	0.086	0.089	0.945	0.344	-0.092	0.263
				Distance to river	0.14	-0.085	0.092	0.909	0.363	-0.269	0.098

Lichens

Species group	Random term	M	K	Fixed effect	RVI	Estimate	SE	z-value	*p*	2.5% CI	97.5% CI
**All species**	(1|Site/Ad.trees)	11	6								
				No.trees200m	1.00	0.141	0.039	3.539	0.000 ***	0.063	0.219
				DBH	0.41	-0.054	0.037	1.449	0.147	-0.127	0.019
				Altitude	0.52	0.089	0.043	2.020	0.043 *	0.003	0.175
				Crown volume	0.31	-0.043	0.038	1.123	0.262	-0.118	0.032
				Distance to river	0.20	-0.039	0.040	0.962	0.336	-0.120	0.041
				Radiation	0.06	-0.026	0.033	0.769	0.442	-0.091	0.040
**Red-listed**	(1|Site/Ad.trees)	5	6								
				DBH	1.00	-0.760	0.213	3.517	0.000 ***	-1.183	-0.336
				Phen.age2	1.00	0.377	0.222	1.673	0.094 #	-0.065	0.818
				Phen.age3		1.054	0.346	3.005	0.003 **	0.366	1.742
				No.trees200m	1.00	0.438	0.174	2.487	0.013 *	0.093	0.783
				Pot.habitat50m	0.40	-0.281	0.194	1.425	0.154	-0.666	0.105
				Altitude	0.26	-0.120	0.138	0.859	0.391	-0.395	0.154
				Radiation	0.13	-0.100	0.138	0.719	0.472	-0.374	0.173
**Not red-listed**	(1|Site/Ad.trees)	8	7								
				Altitude	0.78	0.104	0.037	2.758	0.006 **	0.030	0.177
				No.trees200m	1.00	0.113	0.039	2.891	0.004 **	0.036	0.189
				DBH	0.17	-0.049	0.035	1.384	0.166	-0.119	0.020
				Crown volume	0.15	-0.046	0.035	1.287	0.198	-0.116	0.024
				Pot.habitat50m	0.09	0.041	0.047	0.854	0.393	-0.053	0.136
				Distance to river	0.17	-0.036	0.040	0.872	0.383	-0.116	0.045
				Radiation	0.08	-0.020	0.034	0.575	0.566	-0.086	0.047
**Small diaspores**	(1|Site/Ad.trees)	5	5								
				DBH	0.65	-0.074	0.041	1.776	0.076 #	-0.155	0.008
				Distance to river	0.79	-0.076	0.041	1.850	0.064 #	-0.158	0.005
				No.trees200m	1.00	0.108	0.045	2.363	0.018 *	0.018	0.198
				Crown volume	0.36	-0.058	0.044	1.321	0.187	-0.145	0.028
**Large diaspores**	(1|Site/Ad.trees)	2	5								
				Altitude	1.00	0.394	0.088	4.414	0.000 ***	0.219	0.569
				Distance to river	1.00	0.234	0.095	2.411	0.016 *	0.044	0.423
				Phen.age2	1.00	0.370	0.144	2.538	0.011 *	0.084	0.656
				Phen.age3		0.540	0.198	2.693	0.007 **	0.147	0.933
				No.trees200m	1.00	0.271	0.088	3.038	0.002 **	0.096	0.446
				DBH	0.35	-0.132	0.118	1.103	0.270	-0.366	0.103
**Non-epiphytes**	(1|Site/Ad.trees)	12	7								
				DBH	0.74	0.334	0.137	2.398	0.017 *	0.061	0.606
				Altitude	0.95	0.383	0.150	2.522	0.012 *	0.085	0.680
				Distance to river	0.71	0.287	0.143	1.977	0.048 *	0.002	0.572
				Phen.age2	0.33	0.462	0.203	2.247	0.025 *	0.059	0.865
				Phen.age3		0.604	0.268	2.222	0.026 *	0.071	1.136
				Radiation	0.23	-0.152	0.120	1.254	0.210	-0.390	0.086
				Crown volume	0.16	-0.170	0.140	1.195	0.232	-0.448	0.109
				No.trees200m	0.13	0.169	0.156	1.069	0.285	-0.141	0.480
**Epiphytes**	(1|Site/Ad.trees)	4	4								
				DBH	1.00	-0.086	0.038	2.254	0.024 *	-0.161	-0.011
				Altitude	0.49	0.081	0.035	2.260	0.024 *	0.011	0.152
				No.trees200m	1.00	0.137	0.040	3.413	0.001 ***	0.058	0.216
				Distance to river	0.39	-0.046	0.039	1.170	0.242	-0.124	0.031
**- Red-listed**	(1|Site/Ad.trees)	5	6								
				DBH	1.00	-0.760	0.213	3.517	0.000 ***	-1.183	-0.336
				Phen.age2	1.00	0.377	0.222	1.673	0.094 #	-0.065	0.818
				Phen.age3		1.054	0.346	3.005	0.003 **	0.366	1.742

				No.trees200m	1.00	0.438	0.174	2.487	0.013	*	0.093	0.783
				Pot.habitat50m	0.40	-0.281	0.194	1.425	0.154		-0.666	0.105
				Altitude	0.26	-0.120	0.138	0.859	0.391		-0.395	0.154
				Radiation	0.13	-0.100	0.138	0.719	0.472		-0.374	0.173
**- Not red-listed**	(1|Site/Ad.trees)	8	5									
				DBH	0.81	-0.076	0.039	1.951	0.051	#	-0.153	0.000
				Altitude	0.55	0.099	0.037	2.661	0.008	**	0.026	0.172
				No.trees200m	0.91	0.113	0.044	2.527	0.012	*	0.025	0.201
				Distance to river	0.34	-0.052	0.041	1.264	0.206		-0.133	0.029
				Pot.habitat50m	0.18	0.065	0.052	1.235	0.217		-0.038	0.169
**- Small diaspores**	(1|Site/Ad.trees)	2	3									
				DBH	1.00	-0.090	0.038	2.298	0.022	*	-0.166	-0.013
				Distance to river	0.65	-0.077	0.041	1.870	0.062	#	-0.158	0.004
				No.trees200m	1.00	0.112	0.045	2.430	0.015	*	0.022	0.202
**- Large diaspores**	(1|Site/Ad.trees)	6	5									
				Altitude	1.00	0.314	0.102	3.026	0.002	**	0.111	0.518
				Distance to river	0.51	0.169	0.108	1.540	0.123		-0.046	0.384
				No.trees200m	1.00	0.346	0.102	3.354	0.001	***	0.144	0.548
				DBH	0.31	-0.245	0.140	1.727	0.084	#	-0.524	0.033
				Phen.age2	0.52	0.296	0.172	1.704	0.088	#	-0.044	0.636
				Phen.age3		0.479	0.268	1.769	0.077	#	-0.052	1.009

Table F. Results of the GLMM analyses determining effects on the occurrence of the three focal species. Random term of the model and for fixed effects standardized coefficient estimates, relative variable importance (RVI), unconditional standard error (SE), z-value, p-value and 95 % confidence intervals (CI) after conditional model averaging are shown for the effects of tree characteristics, environmental variables and isolation measures on the occurrence of *Tayloria rudolphiana*, *Lobaria pulmonaria and Orthotrichum rogeri*. Ad.trees additional trees; ol observational level; M number of models in the candidate model set (Δ AICc <2) considered for averaging estimates; K number of predictors in the candidate model set; DBH diameter at breast height; Phen.age phenological age, 2 mature trees, 3 ancient trees (baseline = young trees); Pot.habitat50m potential habitat amount within radius of 50 m, No.trees200m number of trees within radius of 200 m; # $p < 0.1$, * $p < 0.05$, ** $p < 0.01$, *** $p < 0.001$.

Species	Random term	M	K	Fixed effect	RVI	Estimate	SE	z-value	*p*	2.5% CI	97.5% CI
T. rudolphiana	(1|Site)	5	7								
				Altitude	1.00	2.901	1.356	2.109	0.035 *	0.205	5.596
				Phen.age2	0.64	19.216	4436.5	0.004	0.997	-8806.5	8844.9
				Phen.age3		20.805	4436.5	0.005	0.996	-8804.9	8846.5
				Pot.habitat50m	1.00	2.406	1.135	2.091	0.037 *	0.151	4.661
				Radiation	1.00	-2.134	1.205	1.745	0.081 #	-4.531	0.263
				DBH	0.55	2.185	1.234	1.750	0.080 #	-0.262	4.632
				Crown volume	0.15	0.986	0.927	1.048	0.295	-0.857	2.829
				Distance to river	0.12	-1.212	1.268	0.942	0.346	-3.733	1.309
O. rogeri	(1|Site/Ad.trees)	9	7								
				Distance to river	0.69	1.509	0.843	1.765	0.078 #	-0.167	3.185
				Phen.age2	1.00	-2.719	1.009	2.659	0.008 **	-4.722	-0.715
				Phen.age3		-2.791	1.482	1.861	0.063 #	-5.730	0.149
				Crown volume	0.33	1.160	0.807	1.417	0.156	-0.444	2.765
				DBH	0.21	1.226	0.957	1.263	0.207	-0.677	3.130
				Radiation	0.37	0.920	0.705	1.287	0.198	-0.482	2.322
L. pulmonaria	(1|Site/Ad.trees)	4	5								
				DBH	1.00	-3.545	1.269	2.754	0.006 **	-6.068	-1.022
				Crown volume	1.00	2.467	1.069	2.273	0.023 *	0.340	4.594
				Phen.age2	1.00	21.404	530.8	0.040	0.968	-1034.3	1077.1
				Phen.age3		21.721	530.8	0.040	0.968	-1034.0	1077.5
				Distance to river	0.21	0.853	0.874	0.962	0.336	-0.884	2.591
				No.trees200m	0.20	0.921	1.025	0.885	0.376	-1.117	2.959
				Radiation	0.17	-0.612	0.826	0.730	0.465	-2.254	1.031
